# Metal alkyls programmed to generate metal alkylidenes by α-H abstraction: prognosis from NMR chemical shift†
†Electronic supplementary information (ESI) available: Experimental and computational details, NMR spectra, results of NMR calculations and NCS analysis, graphical representation of shielding tensors, molecular orbital diagrams of selected compounds, optimized structures for all calculated species. See DOI: 10.1039/c7sc05039a


**DOI:** 10.1039/c7sc05039a

**Published:** 2018-01-05

**Authors:** Christopher P. Gordon, Keishi Yamamoto, Keith Searles, Satoru Shirase, Richard A. Andersen, Odile Eisenstein, Christophe Copéret

**Affiliations:** a Department of Chemistry and Applied Biosciences , ETH Zürich , Vladimir Prelog Weg 1-5 , 8093 , Zürich , Switzerland . Email: ccoperet@ethz.ch; b Department of Chemistry , Graduate School of Engineering Science , Osaka University , Toyonaka , Osaka 560-8531 , Japan; c Department of Chemistry , University of California , Berkeley , California 94720 , USA . Email: raandersen@lbl.gov; d Institut Charles Gerhardt , UMR 5253 CNRS-UM-ENSCM , Université de Montpellier , 34095 Montpellier , France . Email: odile.eisenstein@univ-montp2.fr; e Hylleraas Centre for Quantum Molecular Sciences , Department of Chemistry , University of Oslo , P.O. Box 1033, Blindern , 0315 Oslo , Norway

## Abstract

Metal alkylidenes, which are key organometallic intermediates in reactions such as olefination or alkene and alkane metathesis, are typically generated from metal dialkyl compounds [M](CH_2_R)_2_ that show distinctively deshielded chemical shifts for their α-carbons. Experimental solid-state NMR measurements combined with DFT/ZORA calculations and a chemical shift tensor analysis reveal that this remarkable deshielding originates from an empty metal d-orbital oriented in the M–C_α_–C_α′_ plane, interacting with the C_α_ p-orbital lying in the same plane. This π-type interaction inscribes some alkylidene character into C_α_ that favors alkylidene generation *via* α-H abstraction. The extent of the deshielding and the anisotropy of the alkyl chemical shift tensors distinguishes [M](CH_2_R)_2_ compounds that form alkylidenes from those that do not, relating the reactivity to molecular orbitals of the respective molecules. The α-carbon chemical shifts and tensor orientations thus predict the reactivity of metal alkyl compounds towards alkylidene generation.

## Abstract 2

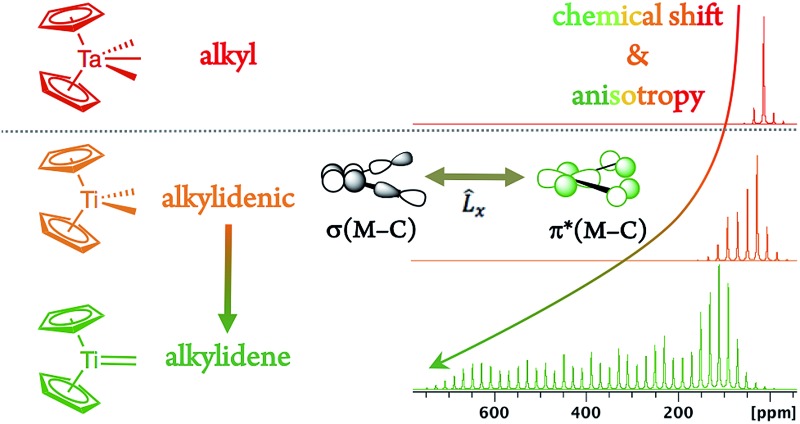
Chemical shift analysis predicts the ease of alkylidene formation from bis-alkyl d^0^ complexes *via* α-H abstraction.

## Introduction

**Scheme 1:**
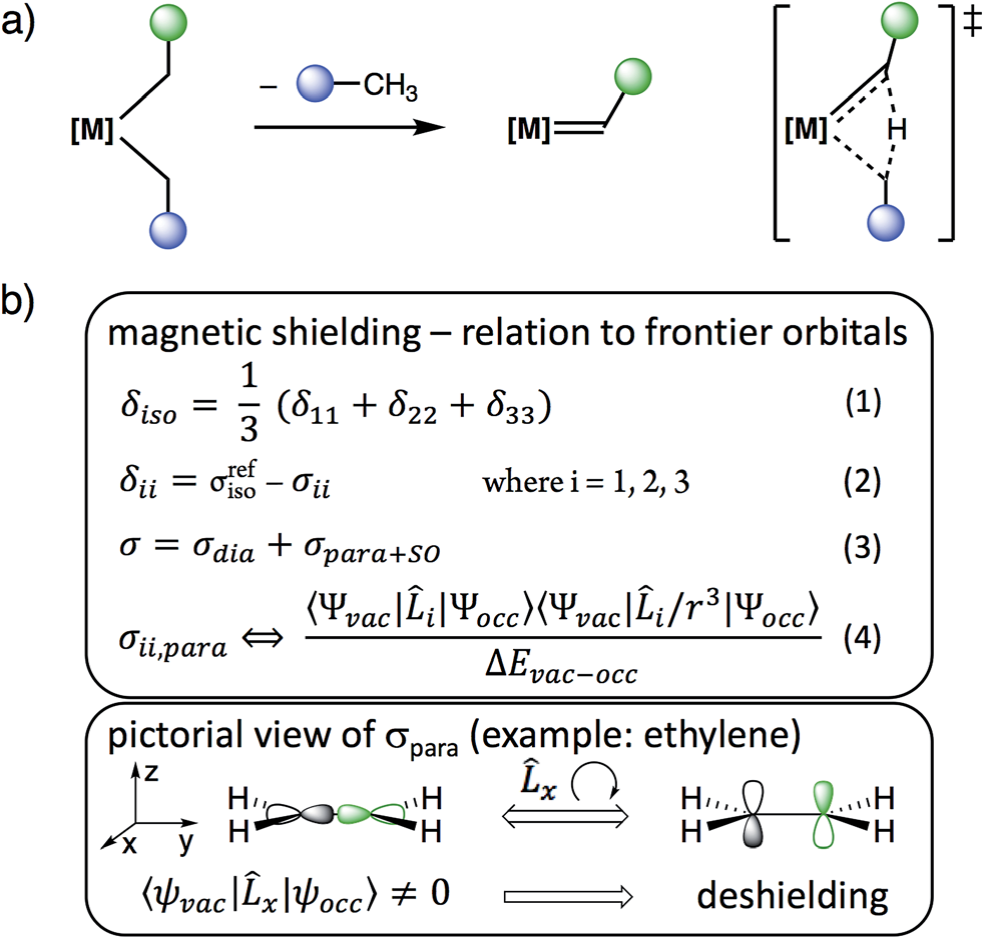
(a) Alkylidene formation from [M](CH_2_R)_2_*via* α-H abstraction and (b) relation between chemical shielding, chemical shift and frontier molecular orbitals.

Metal alkylidenes are key intermediates in many prominent chemical reactions, such as C–H activation, olefination reactions,^1^ and catalytic alkene and alkane metathesis.^2^–^4^ These compounds are commonly generated by deprotonation of a metal alkyl,^5^ carbene transfer or α-H abstraction from [M](CH_2_R)_2_ species.^2^,^6^–^12^ The latter process is particularly favoured for neopentyl (R = *t*Bu) and related ligands, that were originally used to avoid the decomposition of these alkyl compounds *via* β-H transfer.^13^,^14^ These dialkyl compounds can however decompose *via* α-H abstraction, an intramolecular deprotonation process between two *cis*-bound alkyl ligands on a metal centre, typically with d^0^ configuration, related to σ-bond metathesis (Scheme 1a). While ubiquitous and used for the synthesis of numerous alkylidenes, no physical properties are currently available to guide the chemist in deciding which [M](CH_2_R)_2_ fragment will easily generate a [M](=CHR) species *via* α-H abstraction and in understanding why this process occurs.

We reasoned that solid state NMR spectroscopy could be an ideal tool to probe this type of reactivity, since chemical shielding and associated ^13^C chemical shifts (*δ*_iso_ and the principal tensor components *δ*_11_ ≥ *δ*_22_ ≥ *δ*_33_) are directly linked to frontier molecular orbitals that control reactivity (Scheme 1b).^15^ This article reports the experimental measurement, calculation, and orbital analysis of the chemical shift tensor (CST) of the deshielded α-carbons in [M](CH_2_R)_2_ compounds that are prone to yield alkylidenes.

In short, we show that the occurrence of α-H abstraction from metal dialkyl compounds requires the presence of a low-lying empty metal d-orbital that points into the M–C_α_–C_α′_ plane. The signature of this orbital is the distinctively deshielded ^13^C chemical shift of the α-carbons and a specific orientation of the CST, arising from the alkylidene character on the α-carbon and polarization of the C^–*δ*^–H^+*δ*^ bond. This situation is particularly pronounced for neopentyl ligands, explaining their propensity to generate alkylidenes *via* α-H abstraction.

## Results and discussion

**Fig. 1:**
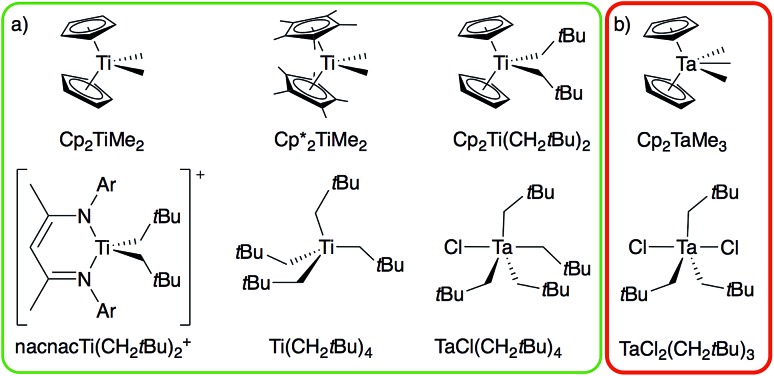
Ti and Ta alkyl compounds that (a) readily form alkylidenes *via* α-H abstraction (green box) or that (b) do not undergo α-H abstraction (red box).

From the broad range of metal alkyl compounds that undergo α-H abstraction to yield well-defined alkylidenes or putative alkylidene species, a set of Ti and Ta compounds is selected as representative examples, chosen for historical reasons and their well-established reactivity patterns (Fig. 1). We focus on the Petasis reagent, Cp_2_Ti(CH_3_)_2_, a well-known olefination agent^16^ involving the putative methylidene intermediate Cp_2_Ti(CH_2_), which is trapped as Cp_2_Ti(CH_2_)(PMe_3_) in the presence of PMe_3_.^17^ We also include the related compound Cp*_2_Ti(CH_3_)_2_,^18^ Cp_2_Ti(CH_2_*t*Bu)_2_,^17^ Ti(CH_2_*t*Bu)_4_,^19^ and the cationic alkyl compound [nacnacTi(CH_2_*t*Bu)_2_]^+^ (nacnac = [Ar]NC(CH_3_)CHC(CH_3_)N[Ar], Ar = 2,6-(CH(CH_3_)_2_)_2_C_6_H_3_), which generate the corresponding neopentylidenes.^20^ We also prepare the d^0^ tantalum compound, TaCl(CH_2_*t*Bu)_4_,^6^ an isolable intermediate in the synthesis of the first well-defined metal alkylidene, Ta(CH_2_*t*Bu)_3_(CH*t*Bu), which cleanly transforms into the corresponding alkylidene TaCl(CH_2_*t*Bu)_2_(CH*t*Bu); analogous to the decomposition of Ta(CH_2_*t*Bu)_5_ into Ta(CH_2_*t*Bu)_3_(CH*t*Bu).^21^,^22^ We also study TaCl_2_(CH_2_*t*Bu)_3_ 23,24 and Cp_2_Ta(CH_3_)_3_, which do not generate the corresponding alkylidenes (see Fig. 1b, red box), and the related cationic compound [Cp_2_Ta(CH_3_)_2_][BF_4_].^25^ These organometallic compounds provide an experimental test to distinguish between those metal dialkyl compounds that do and those that do not form alkylidenes.

The structures of the studied metal alkyl compounds are shown in Fig. 1 and the measured and calculated chemical shift tensors are reported in Table 1. The isotropic chemical shift *δ*_iso_, observed in solution NMR, is the average value of the three principal tensor components (*δ*_11_ ≥ *δ*_22_ ≥ *δ*_33_) obtained by solid-state NMR (Scheme 1b, eqn (1)).

**Table 1:** Measured α-carbon chemical shift tensors in ppm of selected metal alkyls and related alkylidenes. The calculated values are given in parenthesis

Compound	*δ*_iso_	*δ*_11_	*δ*_22_	*δ*_33_
Cp_2_Ti(CH_3_)_2_	52 (52)	121 (118)	31 (35)	3 (2)
Cp*_2_Ti(CH_3_)_2_	50 (51)	100 (114)	30 (26)	22 (12)
Cp_2_Ti(CH_2_*t*Bu)_2_	98 (86)	193 (158)	70 (60)	31 (39)
Cp_2_Ti(CH_2_)-PMe_3_	286 (309)	714 (754)	82 (155)	70 (17)
nacnacTi(CH_2_*t*Bu)_2_^+^	143 (139)	217 (212)	184 (175)	28 (30)
nacnacTi(CH*t*Bu)(OTf)	271 (277)	569 (613)	271 (265)	–28 (–47)
Ti(CH_2_*t*Bu)_4_	119 (118)	165 (158)	137 (148)	55 (49)
TaCl(CH_2_*t*Bu)_4_^*a*^	135 (142)	214 (214)	149 (147)	43 (64)
TaCl(CH_2_*t*Bu)_4_^*b*^	113 (126)	154 (159)	146 (162)	40 (58)
TaCl(CH_2_*t*Bu)_2_(CH*t*Bu)	251 (264)	—^*e*^ (484)	—^*e*^ (310)	—^*e*^ (–2)
TaCl_2_(CH_2_*t*Bu)_3_	115 (130)	156 (171)	141 (152)	48 (65)
Cp_2_Ta(CH_3_)_3_^*c*^	25 (22)	43 (37)	28 (33)	4 (–4)
Cp_2_Ta(CH_3_)_3_^*d*^	22 (23)	48 (49)	19 (15)	–1 (7)
[Cp_2_Ta(CH_3_)_2_][BF_4_]	57 (62)	172 (164)	24 (42)	–27 (–20)

^*a*^Axial,

^*b*^Equatorial,

^*c*^External and

^*d*^Internal carbons.

^*e*^Not measured.

**Fig. 2:**
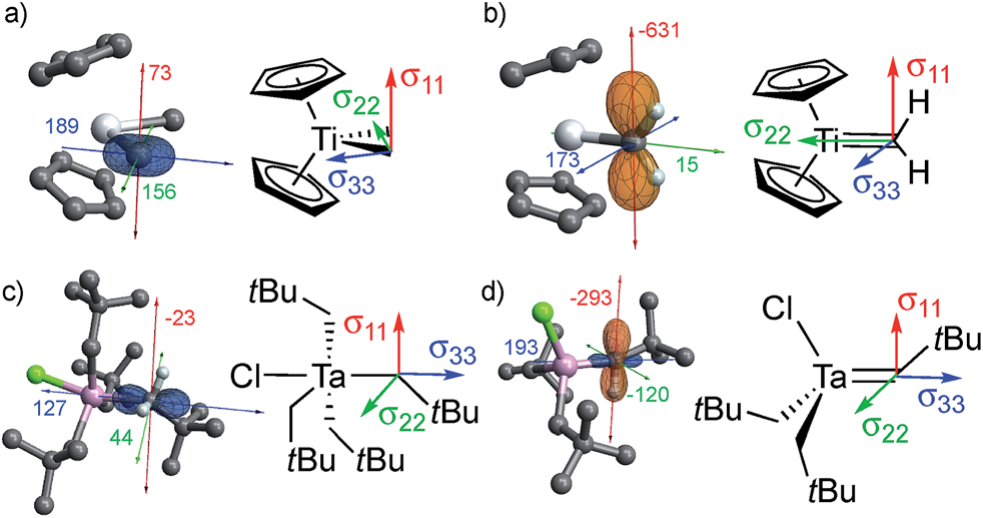
Orientation of the shielding tensor for (a) Cp_2_Ti(CH_3_)_2_, (b) Cp_2_Ti(CH_2_), (c) TaCl(CH_2_*t*Bu)_4_ and (d) TaCl(CH_2_*t*Bu)_2_(CHtBu). The shielding tensor is oriented similarly both in metal alkyls and the corresponding alkylidenes. The latter are more strongly deshielded, mainly due to their highly deshielded *σ*_11_ component (see text); the chemical shift and shielding are directly related by eqn (2). In these images, orange lobes show deshielded regions (*σ* < 0), while blue lobes show shielded regions (*σ* > 0).

All of the metal alkyl compounds, with the exception of Cp_2_Ta(CH_3_)_3_, display unusually large spans (*Ω* = *δ*_11_ – *δ*_33_) for the α-alkyl-carbons with deshielded *δ*_iso_. The low-field value of *δ*_iso_ is mostly due to the strongly deshielded *δ*_11_ component. The experimental values are compared to those obtained by DFT/ZORA calculations (see ESI† for Computational Details), which also provide the tensor orientation. The obtained shieldings and associated chemical shifts reproduce well the experimental isotropic values and show good agreements with the individual tensor components (Table 1). The calculated shielding tensors are shown for specific examples in Fig. 2 (the tensors of the other compounds are shown in Fig. S14†).

For Cp_2_Ti(CH_3_)_2_, the most deshielded component (*δ*_11_) is oriented perpendicular to the plane that contains the two M–C_α_ bonds (Fig. 2a). This orientation is the same as in the associated alkylidene, Cp_2_Ti(CH_2_) (Fig. 2b), and the isolated adduct Cp_2_Ti(CH_2_)(PMe_3_), for which the most deshielded component is oriented perpendicular to the *σ*(M=C) and the π(M=C) bonds.^26^ These similarities implicate alkylidene character in the carbon atoms of the methyl groups in Cp_2_Ti(CH_3_)_2_.

The axial carbon in the trigonal bipyramidal (TBP) molecule TaCl(CH_2_*t*Bu)_4_ (Fig. 2c) has the two most deshielded tensor components *δ*_11_ and *δ*_22_ oriented perpendicular to the M–C axis, again with *δ*_11_ being perpendicular to the plane containing two M–C_α_ bonds, while the most shielded component *δ*_33_ is along the M–C axis. The orientation of the tensor in this compound is similar to that of the associated alkylidene (Fig. 2d). It should be noted that TaCl(CH_2_*t*Bu)_4_ shows two distinct signals in the solution NMR at 144 and 116 ppm in a 1 : 3 ratio, corresponding to axial and equatorial CH_2_*t*Bu ligands of the trigonal bipyramidal structure, respectively. In solid-state NMR, the situation is complicated by the presence of several sites in the powdered sample. At 100 K, six distinct sites are observed in a chemical shift range of 113 to 135 ppm (the extreme values are reported in Table 1, for other values see Table S1†). The most deshielded site (135 ppm) stands out by a notably large span (*Ω* = 171 ppm) and is tentatively assigned to the axial carbon, while the other sites show spans (*Ω*) that vary between 102 and 143 ppm and presumably correspond to equatorial α-carbons in different environments. DFT calculations also show that the axial α-carbon is strongly deshielded (*δ*_iso_/*δ*_11_ = 142/214 ppm), while the equatorial carbons are more shielded (*δ*_iso_ = 126–129 ppm, *δ*_11_ = 159–189 ppm). The calculations show the presence of several local minima upon rotation of the axial CH_2_*t*Bu ligand in TaCl(CH_2_*t*Bu)_4_, giving rise to an ensemble of conformations as a possible explanation for the observation of several peaks.

The origin of the deshielded chemical shift values is investigated by an orbital analysis of the corresponding shielding tensor (*σ*, eqn (2)).^15^ Decomposition of the shielding into diamagnetic (*σ*_dia_) and paramagnetic contributions, which also include contributions from spin–orbit coupling (*σ*_*para*+SO_, eqn (3)), reveals that the variation in the shielding values is mostly associated with *σ*_*para*+SO_. For the compounds investigated here, spin–orbit coupling is relatively small (Table S7†), allowing for interpreting *σ*_*para*+SO_ based solely on the paramagnetic contributions. These originate from the magnetically induced coupling of excited electronic states with the ground state, by action of the angular momentum operator *L*_*i*_ (eqn (4)). Hence, the chemical shift is sensitive to the relative energy and orientation of the frontier orbitals, establishing a link to reactivity. For carbon p-orbitals, deshielding along direction *i* occurs when the vacant and occupied orbitals are oriented perpendicular to each other and to the *i*-axis.

**Fig. 3:**
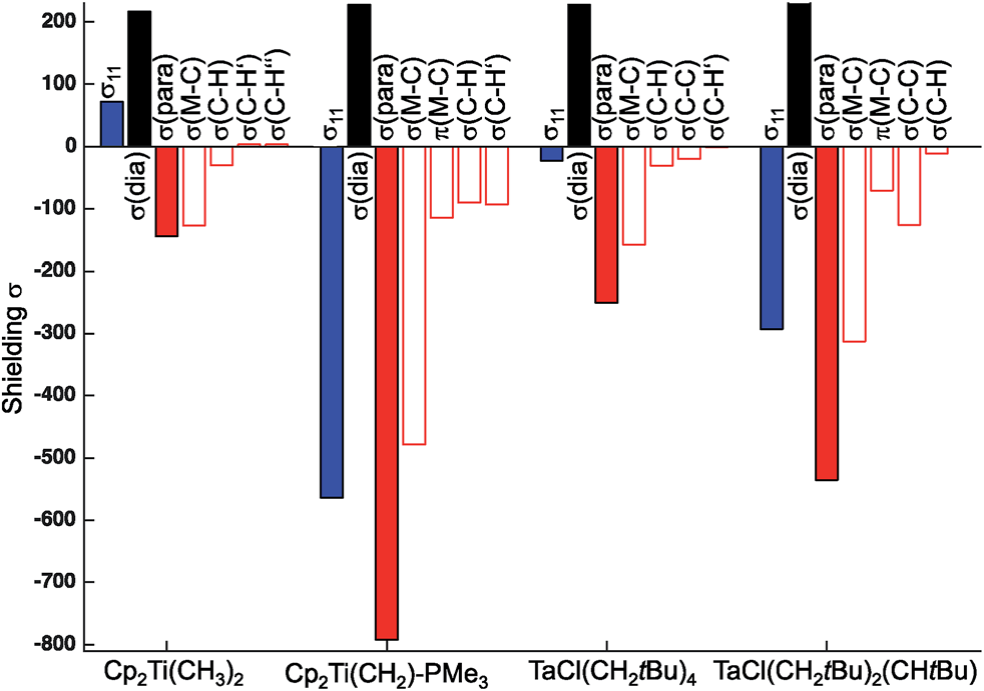
Orbital contributions to *σ*_11_ in Cp_2_Ti(CH_3_)_2_, TaCl(CH_2_*t*Bu)_4_ (axial carbon atom), and associated alkylidenes.

The individual orbital contributions to the most deshielded component of the CST (*δ*_11_/*σ*_11_), obtained through a Natural Chemical Shift (NCS) analysis^26^–^56^ of the representative examples, Cp_2_Ti(CH_3_)_2_ and TaCl(CH_2_*t*Bu)_4_, and the associated alkylidenes are plotted in Fig. 3 (values given in Table S4†). Notably, the largest contribution to deshielding in the *δ*_11_/*σ*_11_ component of the metal alkyl compounds is always associated with the *σ*(M–C_α_) bond, as found for the corresponding metal alkylidenes.

**Fig. 4:**
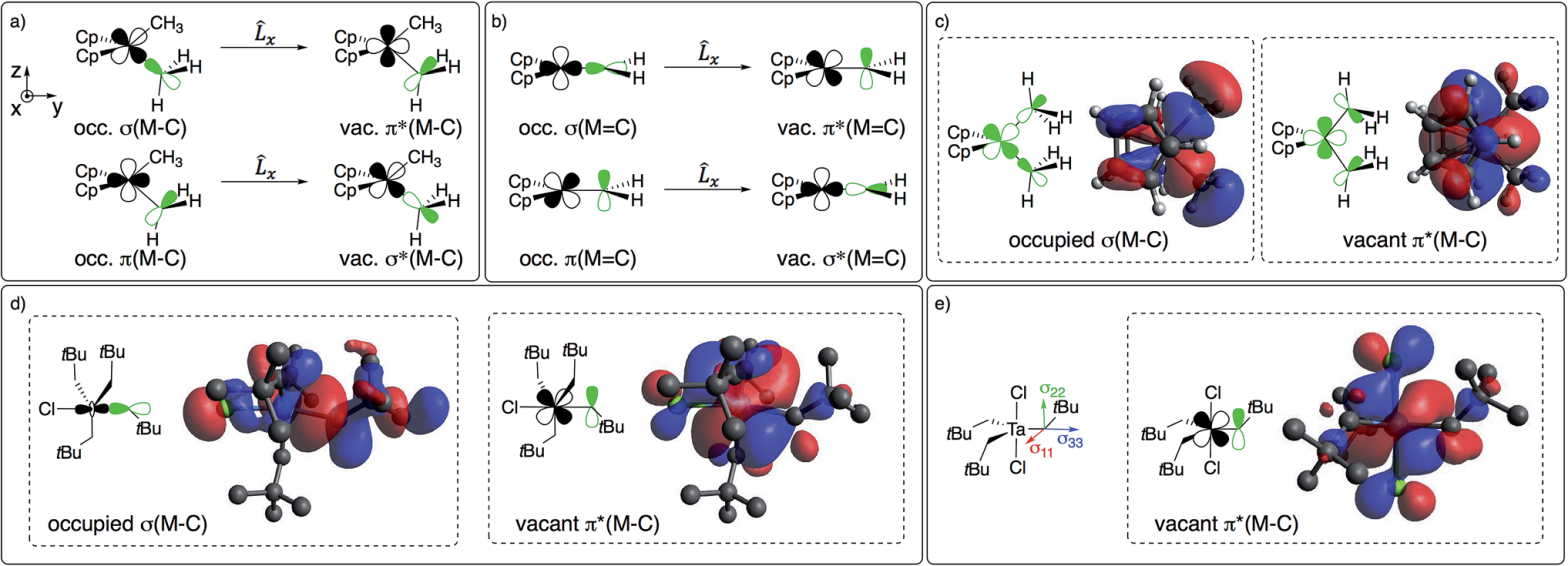
(a) Main orbital contributions to the paramagnetic term for *δ*_11_ in Cp_2_M(CH_3_)_2_ and (b) in Cp_2_Ti(CH_2_). Frontier molecular orbitals leading to deshielding in (c) Cp_2_M(CH_3_)_2_ (M = Ti or Ta^+^) and (d) TaCl(CH_2_*t*Bu)_4_ (see Fig. 2 for orientation of CST). (e) CST orientation and LUMO of TaCl_2_(CH_2_*t*Bu)_3_.

The large deshielding of the α-carbons, originating from the *σ*(M–C_α_) bond, indicates the presence of a low-lying vacant orbital that is oriented perpendicular to the M–C axis and the direction of the deshielding. The emergence of this low-lying empty orbital is due to the weak π-donating ability of alkyl groups that, by interaction with an empty d_π_ metal orbital, develop a π-interaction by which the M–CH_2_R bond acquires alkylidene character, as shown for Cp_2_Ti(CH_3_)_2_ in Fig. 4c; this orbital is labelled as π(M–C). The associated π*(M–C) orbital, which is the LUMO of the compound (Fig. 4c right and S15†), is responsible for the observed deshielding by coupling with the occupied *σ*(M–C) orbital (Fig. 4a). In the corresponding alkylidene Cp_2_Ti(CH_2_), there is a smaller energy gap between the *σ*(M=C) and π*(M=C) orbitals, hence a significantly larger deshielding (Fig. 4b). Similarly, in TaCl(CH_2_*t*Bu)_4_ the largest part of the deshielding on the axial α-carbon originates from the occupied *σ*(M–C) orbital (Fig. 4d left), which is coupled to the vacant π*(M–C) orbital (Fig. 4d right), again evidencing a π-type interaction of the metal atom with the alkyl ligand. The larger deshielding of the axial carbon atom as compared to the equatorial carbon atoms indicates larger π-character in the former M–C bond.

A similar pattern and analysis applies to Cp*_2_Ti(CH_3_)_2_, Cp_2_Ti(CH_2_*t*Bu)_2_, nacnacTi(CH_2_*t*Bu)_2_^+^, and Ti(CH_2_*t*Bu)_4_, where deshielding of the α-carbon mostly arises from the *σ*(M–C) bond, indicating a low-lying orbital of π*(M–C) character, oriented perpendicular to the *σ*(M–C) bond (Table S4 and Fig. S11†). The emergence of this orbital requires an empty metal d-orbital in the M–C_α_–C_α′_ plane of the correct symmetry to interact with the p_π_-orbital of the alkyl ligand. Such an orbital is indeed present in all the aforementioned bis-alkyl compounds that generate alkylidenes *via* α-H abstraction (Fig. S15†). Thus, the deshielding is a signature of alkylidenic character in the metal–carbon bond.

The metal neopentyl compounds show particularly low ^1^*J*_C–H_ coupling constants (*e.g.* 116 Hz for Cp_2_Ti(CH_2_*t*Bu)_2_, 105 Hz and 110 Hz for the two α-carbons in nacnacTi(CH_2_*t*Bu)_2_^+^, 110 Hz in Ti(CH_2_*t*Bu)_4_, and 96 Hz for the axial carbon in TaCl(CH_2_*t*Bu)_4_). This effect is slightly less pronounced in Cp*_2_TiMe_2_ (122 Hz average coupling constant). These lowered coupling constants indicate more p-orbital character in the C–H bonds, an additional signature of a π(M–C) type interaction. Notably, the equatorial carbons in TaCl(CH_2_*t*Bu)_4_ show a larger ^1^*J*_C–H_ coupling constant (115 Hz) than the axial carbon (96 Hz), indicating a less developed π-interaction in the former, in line with the less deshielded carbon atoms.

The formation of alkylidenes from bis-alkyl complexes *via* α-H abstraction requires the presence of a low-lying empty orbital in the plane of the M–C_α_ and M–C_α′_ bonds. While the deshielded chemical shift value and large anisotropy of the CST indicate the presence of such an orbital, the CST orientation probes the location of this empty orbital. For example, TaCl_2_(CH_2_*t*Bu)_3_ features a trigonal-bipyramidal geometry with two Cl-ligands in axial positions. The rather large deshielding on the α-carbon (115 ppm) indicates a low-lying empty orbital, but the most deshielded component (*δ*_11_) of the CST is not perpendicular to a plane containing two equatorial Ta–C_α_ bonds but rather perpendicular to the plane containing an equatorial Ta–C_α_ and an axial Ta–Cl bond, which is also the plane containing the LUMO (Fig. 4e). Accordingly, the alkylidenic character is not developed in the direction needed for α-H abstraction. This compound is therefore stable, even when heated to 100 °C in the presence of PMe_3_ for 4 h, in contrast to TaCl(CH_2_*t*Bu)_4_. In other words, in a trigonal-bipyramidal structure, α-H abstraction is favoured between an axial and an equatorial alkyl ligand and is not readily accessible in TaCl_2_(CH_2_*t*Bu)_3_, where both axial positions are occupied by Cl-ligands.

**Fig. 5:**
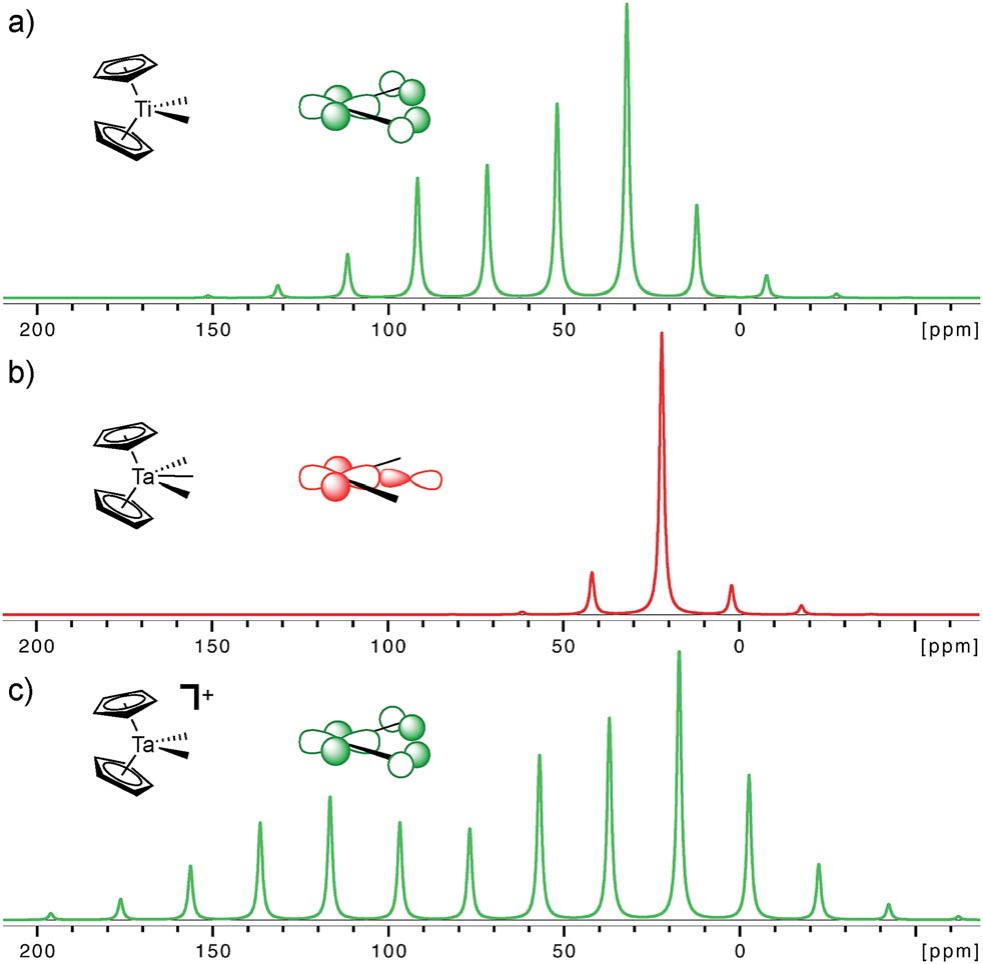
Simulated solid state NMR spectra for the α-carbons in Cp_2_Ti(CH_3_)_2_, Cp_2_Ta(CH_3_)_3_ (external carbon atom) and [Cp_2_Ta(CH_3_)_2_]^+^ at a magic angle spinning frequency of 2 kHz. The inserted molecular orbitals show the presence (a and c) and the absence (b) of the key empty orbital that leads to alkylidene character. The experimental solid state NMR spectra are available in the ESI.†

The importance of the presence of a vacant metal d-orbital with the appropriate orientation for the observed deshielding is further demonstrated by comparing Cp_2_Ti(CH_3_)_2_, Cp_2_Ta(CH_3_)_3_, and Cp_2_Ta(CH_3_)_2_^+^. While Cp_2_Ti(CH_3_)_2_ and Cp_2_Ta(CH_3_)_2_^+^ both show rather deshielded and anisotropic α-carbons, the NMR signatures of Cp_2_Ta(CH_3_)_3_ are markedly different (Fig. 5). The chemical shift drops from 52 ppm in Cp_2_Ti(CH_3_)_2_ to 22 and 25 ppm in Cp_2_Ta(CH_3_)_3_ (external and internal carbons, respectively), mostly originating from a large decrease of the *δ*_11_ component (121 ppm in Cp_2_Ti(CH_3_)_2_ as compared to <50 ppm for Cp_2_Ta(CH_3_)_3_). The third methyl-substituent in Cp_2_Ta(CH_3_)_3_ interacts with the empty metal orbital that is required for developing the alkylidene character (the remaining empty orbitals on the metal, which are involved in bonding with the Cp rings, are too high in energy for such an interaction). However, abstraction of a methyl-ligand generates Cp_2_Ta(CH_3_)_2_^+^, isoelectronic to Cp_2_Ti(CH_3_)_2_, and restores the highly anisotropic CST and deshielded chemical shift values (Fig. 5 and Table 1).

**Fig. 6:**
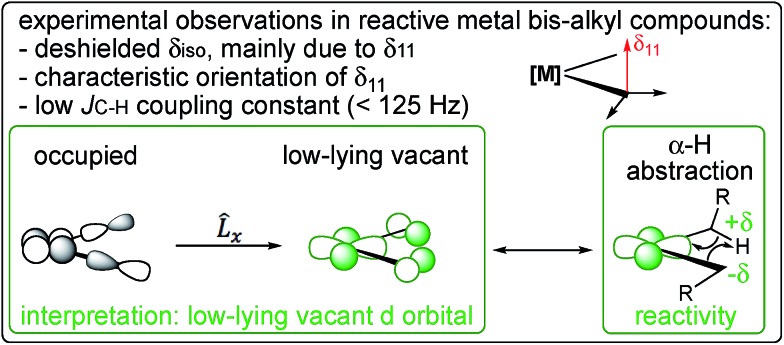
NMR chemical shift and ^1^*J*_C–H_ coupling constant are signatures of a low-lying empty metal d-orbital that leads to a partial π(M–C) interaction and favours α-H abstraction.

The nature of the alkyl ligands also plays an important role in manipulating the alkylidene character on the α-carbon. This is illustrated by comparing the bis-neopentyl metallocene, Cp_2_Ti(CH_2_*t*Bu)_2_ with the bis-methyl metallocenes, Cp_2_Ti(CH_3_)_2_ and Cp*_2_Ti(CH_3_)_2_, since detailed kinetic data on the decomposition *via* α-H abstraction is available for the latter metallocene.^57^ Cp_2_Ti(CH_2_*t*Bu)_2_ displays a much more deshielded and anisotropic α-carbon, due to the coupling of *σ*(M–C) with π*(M–C), in addition to a significant contribution of the coupling of *σ*(C_α_–C_β_) and π*(M–C_α_) (Table S4 and Fig. S11†). The contribution of *σ*(C_α_–C_β_) is associated with the wide calculated α(M–C_α_–C_β_) angle of 136°, signalling the increased alkylidene character in the Ti–C bond. The NMR data thus suggest a lower activation energy for α-H abstraction in Cp_2_Ti(CH_2_*t*Bu)_2_, which is confirmed by the calculated energy profiles. The calculated Gibbs activation (and associated reaction) energies at 298 K are +27.1 (–3.3) kcal mol^–1^ for Cp_2_Ti(CH_2_*t*Bu)_2_*vs.* +30.8 (+8.2) kcal mol^–1^ and +32.1 (+5.3) kcal mol^–1^ for Cp_2_Ti(CH_3_)_2_ and Cp*_2_Ti(CH_3_)_2_, respectively. The lower calculated Gibbs activation energy for the α-H abstraction in the neopentyl derivative is consistent with the experimentally determined values found for Cp_2_Ti(CH_2_*t*Bu)_2_ (+22.8 kcal mol^–1^)^58^ and Cp*_2_Ti(CH_3_)_2_ (+28.3 kcal mol^–1^ with *k*_α(H)_/*k*_α(D)_ = 2.92 ± 0.10).^57^ The Gibbs activation energies correlate with the deshielded α-carbon chemical shift and the lower value of the ^1^*J*_C–H_ coupling constant, consistent with more alkylidene character in the Ti–C bond and consequently a lower transition state energy for the α-H abstraction step (Fig. 6). Detailed kinetic studies have also been reported on the elimination of CH_3_*t*Bu from Ti(CH_2_*t*Bu)_4_ 19 and Ta(CH_2_*t*Bu)_5_.^21^,^22^ Both compounds follow first order kinetics for α-H abstraction. For Ti(CH_2_*t*Bu)_4_, values of Δ*G*^‡^ = 26.4 kcal mol^–1^ and *k*_α(H)_/*k*_α(D)_ = 5.2 ± 0.4 were found. For Ta(CH_2_*t*Bu)_5_ the values were Δ*G*^‡^ = 22.3 kcal mol^–1^ and *k*_α(H)_/*k*_α(D)_ = 14.1 ± 0.8 (note that Δ*G*^‡^ was determined for the deuterated compound).

It is important to note the close analogy between alkylidenic character of the M–C bond and the occurrence of what is referred to as α-H agostic interactions.^59^,^60^ α-H Agostic interactions are evidenced by acute M–C–H angles (<109.47°) and are generally associated with low ^1^*J*_C–H_ coupling constants for the carbon bound to the metal (<125 Hz). The presence of an α-H agostic interaction is associated with a π-type interaction of a p-orbital on the α-carbon with a vacant metal d-orbital, resulting in the observed geometrical features and lower ^1^*J*_C–H_ coupling constants.^61^,^62^ Thus, an α-H agostic interaction is also an indirect reporter of an alkylidenic character in a M–C bond. However, the CST values that signal alkylidenic character can be present in the absence of geometrical features or lowered ^1^*J*_C–H_ coupling constants associated with α-H agostic interactions. For example, in TaCl(CH_2_*t*Bu)_4_, one M–C–H bond angle at the axial carbon is calculated to be 95° (which can be considered agostic), whereas, in Cp_2_Ti(CH_3_)_2_, the M–C–H angles are calculated to be 112.7° (the H atom in the *σ*_h_ plane) and 109.7° (the H atom out of *σ*_h_ plane). While both TaCl(CH_2_*t*Bu)_4_ and Cp_2_Ti(CH_3_)_2_ have alkylidenic character, as evidenced by their CSTs, only the former is considered to be α-C–H agostic based on its calculated structure. Our view is that an α-C–H agostic interaction is better described as a π-donation from the carbon p-orbital, rather than as a 3-center-2-electron bond. The philosophical question remains: is the M···H–C_α_ interaction due to the alkylidenic character of the carbon atom bound to the metal, or does the alkylidenic character arise from the M···H–C_α_ interaction? Perhaps a distinction without a difference.

## Conclusions

In summary, strongly deshielded chemical shift values of the α-carbons in [M](CH_2_R)_2_ compounds in combination with a large anisotropy and specific orientation of the chemical shift tensor reveal the presence and location of low-lying empty metal d-orbitals. The alkylidenic character in the M–CH_2_R bond activates the α-C–H bond towards α-H abstraction when the low-lying empty orbital is appropriately oriented. While this orbital arrangement can lead to the development of an α-C–H “agostic” interaction, the magnitude and orientation of the CST is a much more unequivocal signature of the alkylidenic character of the M–C bond. The CST shows that the parent alkyl compounds already contain inscribed information about the reaction products and are programmed to evolve into metal alkylidenes, a situation particularly favoured for neopentyl-type ligands. While this study has focused on Ti and Ta d^0^ compounds, the associated principle is likely applicable to a wide range of metal alkyls with low d-electron counts. The theme of this article is that NMR chemical shift values of atoms directly bonded to a metal centre provide information about the electronic structure and are powerful reporters of the location, orientation, and relative energy of the frontier molecular orbitals. This study shows that chemical shifts can be of predictive value of a compound's reactivity, making their physical interpretation an invaluable tool for the development and the understanding of mechanisms and reactivity. We are currently further exploring this connection.

## Conflicts of interest

There are no conflicts to declare.

## Supplementary Material

Supplementary information

Supplementary information

Supplementary information

Supplementary information

Supplementary information

Supplementary information

Supplementary information

Supplementary information

Supplementary information

Supplementary information

Supplementary information

Supplementary information

Supplementary information

Supplementary information

Supplementary information

Supplementary information

Supplementary information

Supplementary information

Supplementary information

Supplementary information

## References

[cit1] Payack J. F., Huffman M. A., Cai D., Hughes D. L., Collins P. C., Johnson B. K., Cottrell I. F., Tuma L. D. (2004). Org. Process Res. Dev..

[cit2] Schrock R. R. (2009). Chem. Rev..

[cit3] Basset J.-M., Copéret C., Soulivong D., Taoufik M., Cazat J. T. (2010). Acc. Chem. Res..

[cit4] Copéret C. (2010). Chem. Rev..

[cit5] Schrock R. R. (1975). J. Am. Chem. Soc..

[cit6] Schrock R. R. (1974). J. Am. Chem. Soc..

[cit7] Schrock R. R. (1979). Acc. Chem. Res..

[cit8] Schrock R. R. (2005). Chem. Commun..

[cit9] Mindiola D. J. (2006). Acc. Chem. Res..

[cit10] Xue Z.-L., Morton L. A. (2011). J. Organomet. Chem..

[cit11] Rascón F., Copéret C. (2011). J. Organomet. Chem..

[cit12] Schrock R. R., Copéret C. (2017). Organometallics.

[cit13] Wilkinson G. (1974). Science.

[cit14] Schrock R. R., Parshall G. W. (1976). Chem. Rev..

[cit15] Widdifield C. M., Schurko R. W. (2009). Concepts Magn. Reson., Part A.

[cit16] Petasis N. A., Bzowej E. I. (1990). J. Am. Chem. Soc..

[cit17] Meinhart J. D., Anslyn E. V., Grubbs R. H. (1989). Organometallics.

[cit18] Brintzinger H., Bercaw J. E. (1971). J. Am. Chem. Soc..

[cit19] Cheon J., Rogers D. M., Girolami G. S. (1997). J. Am. Chem. Soc..

[cit20] Basuli F., Bailey B. C., Watson L. A., Tomaszewski J., Huffman J. C., Mindiola D. J. (2005). Organometallics.

[cit21] Li L., Hung M., Xue Z. (1995). J. Am. Chem. Soc..

[cit22] Abbott J. K. C., Li L., Xue Z.-L. (2009). J. Am. Chem. Soc..

[cit23] Mowat W., Wilkinson G. (1972). J. Organomet. Chem..

[cit24] Schrock R. R., Fellmann J. D. (1978). J. Am. Chem. Soc..

[cit25] Schrock R. R., Sharp P. R. (1978). J. Am. Chem. Soc..

[cit26] Gordon C. P., Yamamoto K., Liao W.-C., Allouche F., Andersen R. A., Copéret C., Raynaud C., Eisenstein O. (2017). ACS Cent. Sci..

[cit27] Ruiz-Morales Y., Schreckenbach G., Ziegler T. (1996). J. Phys. Chem..

[cit28] Bohmann J. A., Weinhold F., Farrar T. C. (1997). J. Chem. Phys..

[cit29] Salzmann R., Kaupp M., McMahon M. T., Oldfield E. (1998). J. Am. Chem. Soc..

[cit30] Autschbach J. (2008). J. Chem. Phys..

[cit31] Vummaleti S. V. C., Nelson D. J., Poater A., Gomez-Suarez A., Cordes D. B., Slawin A. M. Z., Nolan S. P., Cavallo L. (2015). Chem. Sci..

[cit32] Halbert S., Copéret C., Raynaud C., Eisenstein O. (2016). J. Am. Chem. Soc..

[cit33] Yamamoto K., Gordon C. P., Liao W.-C., Copéret C., Raynaud C., Eisenstein O. (2017). Angew. Chem., Int. Ed..

[cit34] Aquino F., Pritchard B., Autschbach J. (2012). J. Chem. Theory Comput..

[cit35] Wu G., Rovnyak D., Johnson M. J. A., Zanetti N. C., Musaev D. G., Morokuma K., Schrock R. R., Griffin R. G., Cummins C. C. (1996). J. Am. Chem. Soc..

[cit36] Sceats E. L., Figueroa J. S., Cummins C. C., Loening N. M., Van der Wel P., Griffin R. G. (2004). Polyhedron.

[cit37] Greco J. B., Peters J. C., Baker T. A., Davis W. M., Cummins C. C., Wu G. (2001). J. Am. Chem. Soc..

[cit38] Wiberg K. B., Hammer J. D., Zilm K. W., Cheeseman J. R., Keith T. A. (1998). J. Phys. Chem. A.

[cit39] Wiberg K. B., Hammer J. D., Zilm K. W., Cheeseman J. R. (1999). J. Org. Chem..

[cit40] Auer D., Strohmann C., Arbuznikov A. V., Kaupp M. (2003). Organometallics.

[cit41] Auer D., Kaupp M., Strohmann C. (2004). Organometallics.

[cit42] Auer D., Kaupp M., Strohmann C. (2005). Organometallics.

[cit43] Gessner V. H., Meier F., Uhrich D., Kaupp M. (2013). Chem.–Eur. J..

[cit44] Greif A. H., Hrobarik P., Kaupp M. (2017). Chem.–Eur. J..

[cit45] Karni M., Apeloig Y., Takagi N., Nagase S. (2005). Organometallics.

[cit46] Kravchenko V., Kinjo R., Sekiguchi A., Ichinohe M., West R., Balazs Y. S., Schmidt A., Karni M., Apeloig Y. (2006). J. Am. Chem. Soc..

[cit47] Rossini A. J., Mills R. W., Briscoe G. A., Norton E. L., Geier S. J., Hung I., Zheng S., Autschbach J., Schurko R. W. (2009). J. Am. Chem. Soc..

[cit48] Epping J. D., Yao S., Karni M., Apeloig Y., Driess M. (2010). J. Am. Chem. Soc..

[cit49] Standara S., Bouzkova K., Straka M., Zacharova Z., Hocek M., Marek J., Marek R. (2011). Phys. Chem. Chem. Phys..

[cit50] Toušek J., Straka M., Sklenář V., Marek R. (2013). J. Phys. Chem. A.

[cit51] Novotný J., Vicha J., Bora P. L., Repisky M., Straka M., Komorovsky S., Marek R. (2017). J. Chem. Theory Comput..

[cit52] Zhu J., Kurahashi T., Fujii H., Wu G. (2012). Chem. Sci..

[cit53] Pascual-Borras M., Lopez X., Rodriguez-Fortea A., Errington R. J., Poblet J. M. (2014). Chem. Sci..

[cit54] Marchione D., Izquierdo M. A., Bistoni G., Havenith R. W. A., Macchioni A., Zuccaccia D., Tarantelli F., Belpassi L. (2017). Chem.–Eur. J..

[cit55] Lam E., Comas-Vives A., Copéret C. (2017). J. Phys. Chem. C.

[cit56] Estes D. P., Gordon C. P., Fedorov A., Liao W. C., Ehrhorn H., Bittner C., Zier M. L., Bockfeld D., Chan K. W., Eisenstein O., Raynaud C., Tamm M., Copéret C. (2017). J. Am. Chem. Soc..

[cit57] McDade C., Green J. C., Bercaw J. E. (1982). Organometallics.

[cit58] van der Heijden H., Hessen B. (1995). J. Chem. Soc., Chem. Commun..

[cit59] Grubbs R. H., Coates G. W. (1996). Acc. Chem. Res..

[cit60] Brookhart M., Green M. L. H., Parkin G. (2007). Proc. Natl. Acad. Sci. U. S. A..

[cit61] Eisenstein O., Jean Y. (1985). J. Am. Chem. Soc..

[cit62] Albright T. A., Burdett J. K. and Whangbo M.-H., Orbital Interactions in Chemistry, Wiley VCH, Weinheim, 2nd edn, 2013.

