# Supplementary material for: Metal alkyls programmed to generate metal alkylidenes by α-H abstraction: prognosis from NMR chemical shift
Source: Chem Sci. 2018 Jan 5;9(7):1912–8. doi: 10.1039/c7sc05039a (PMC5890791; doi:10.1039/c7sc05039a)
Supplement: Supplementary file 20 [file SC-009-C7SC05039A-s020.pdf]

Electronic Supplementary Material (ESI) for Chemical Science.
This journal is © The Royal Society of Chemistry 2018

Supporting Information

For

Metal Alkyls Programmed to Generate Metal Alkylidenes by α-H Abstraction: Prognosis from NMR Chemical Shift

Christopher P. Gordon,[a,†] Keishi Yamamoto,[a,†] Keith Searles,[a] Satoru Shirase,[a,b] Richard A. Andersen,[c,*] Odile Eisenstein,[d,e,*] Christophe Copéret[a,*]

[a] ETH Zürich, Department of Chemistry and Applied Sciences, Vladimir Prelog Weg 1-5, 10, 8093 Zürich, Switzerland
[b] Department of Chemistry, Graduate School of Engineering Science, Osaka University, Toyonaka, Osaka 560-8531, Japan
[c] Department of Chemistry, University of California, Berkeley, California 94720, United States
[d] Institut Charles Gerhardt, UMR 5253 CNRS-UM-ENSCM, Université de Montpellier, 34095 Montpellier, France
[e] Hylleraas Centre for Quantum Molecular Sciences, Department of Chemistry, University of Oslo, P.O. Box 1033, Blindern, 0315 Oslo, Norway

Contents

1. Experimental section **2**
2. Computational Details **2**
4. NMR Calculations **13**
5. Graphical Representation of the Results of the NCS Analysis **16**
6. Graphical Representations of the Calculated Shielding Tensors **19**
7. MO Diagrams of Representative Metal Alkyl Compounds **21**
8. Optimized Structures of all Calculated Species **21**
9. References **22**

1. Experimental section

General

All experiments involving air- and moisture-sensitive compounds were performed under argon by using standard Schlenk techniques or argon-filled gloveboxes. Pentane, toluene, and diethyl ether were purified using a double MBraun SPS alumina column, and were degassed using three freeze-pump-thaw cycles before use. THF, C_6H_6, C_6D_6, and toluene-d_8 were distilled from Na/Benzophenone. Solution ^1H, and ^{13}C NMR spectra were recorded on Bruker DRX 200, DRX 300, and Avance 400 spectrometers. The magnetic fields were referenced by the deuterium signal of the d-solvent used. The ^1H and ^{13}C spectra were additionally referenced setting the chemical shifts of the residual C_6H_6 signal in C_6D_6 at 7.16 and 128.1 ppm, respectively. $^1J_{C–H}$ coupling constants were determined at room temperature on a Bruker Avance 400 spectrometer by non-decoupled HSQC measurements (solvent: CD_2Cl_2 for [(nacnac)Ti(CH_2tBu)$_2$][BArF$_{24}$] and C_6D_6 for all other compounds).
The solid-state ^1H and ^{13}C NMR spectra were obtained on Bruker Avance III 400, 600, and 700 MHz spectrometers, using a 2.5, 3.2, or 4 mm probe, and the magnetic fields were externally referenced by setting the downfield ^{13}C signal of adamantane to 38.4 ppm. The samples were loaded in a 2.5 or 4 mm zirconia rotor closed with a VESPEL drive cap, or in a 3.2 mm sapphire rotor closed with a zirconia cap with a Teflon insert placed between the sample and the cap to prevent sample spill. Cross polarization magic angle spinning (CPMAS) and spin echo type experiments were used to measure ^{13}C and ^1H spectra, respectively. The ^1H excitation and decoupling radiofrequency (rf) fields were set to 71 kHz and 100 kHz for 4 mm and 2.5/3.2 mm probe, respectively. For CPMAS measurements, the CP condition was optimized to match the Hartmann-Hahn condition under MAS with minor adjustments to reach the best CP efficiency experimentally.

All compounds were synthesized according to literature procedures: $Cp_2Ti(CH_3)_2$,[1] $Cp^*{}_2Ti(CH_3)_2$ [2] $Cp_2Ti(CH_2tBu)_2$ (10% ^{13}C labeled on the α-carbon) ,[3] nacnacTi(CHtBu)(OTf),[4] $Ti(CH_2tBu)_4$ (20% ^{13}C labeled on the α-carbon),[5] $TaCl(CH_2tBu)_4$,[6] $TaCl_2(CH_2tBu)_3$,[7] $TaCl(CH_2tBu)_2(CHtBu)$,[7] $Cp_2Ta(CH_3)_3$,[8] [$Cp_2Ta(CH_3)_2$][BF_4][8].
[(nacnac)Ti(CH_2tBu)$_2$][BArF$_{24}$] (BArF$_{24}$ = tetrakis[(3,5-trifluoromethyl)phenyl]borate) was prepared by a slight modification of the literature procedure.[4] To a 50 mL Schleck flask containing a 10 mL toluene solution of [(nacnac)Ti(CH_2tBu)$_2$] (402 mg, 0.661 mmol) cooled to -30 °C was added dropwise a 10 mL toluene solution of H(OEt_2)$_2$BArF$_{24}$ (671 mg, 0.663 mmol) via a glass pipette. Orange microcrystalline material slowly began to precipitate from the green solution. After stirring for 1 hour, the orange product was isolated on a medium porosity glass frit and washed with 10 ml of pentane. Drying of the material under reduced pressure yielded [(nacnac)Ti(CH_2tBu)$_2$][BArF$_{24}$] in 26% isolated yield (251 mg, 0.171 mmol).

2. Computational Details

All geometry optimizations were performed with the Gaussian09 package[9] with the PBE0 functional.[10] Ti and Ta were represented by the quasi-relativistic effective core potential (RECP) from the Stuttgart group and the associated basis sets[11]-[13]. The remaining atoms (H, C, N, O, F, P and S) were represented by a triple-ζ pcSseg-2 basis set.[14] NMR calculations were performed within the GIAO framework using ADF 2014[15] with the PBE0 functional and Slater-type basis sets of triple-ζ quality (TZ2P). Relativistic effects were treated by the 2 component zeroth order regular approximation (ZORA).[16]-[20] Analysis of scalar-relativistic natural localized molecular orbitals were done with the NBO 6.0 program.[21] Calculated NMR shielding tensors were analyzed using these scalar-relativistic NLMO[22]-[25], with the exception of [nacnacTi(CHtBu)]OTf, for which no orbital analysis was carried out. The 3D representation of the calculated shielding tensors is obtained as polar plots[26],[27] of functions $\sum_{ij} r_i \sigma_{ij} r_j$, with scaling factors of 0.3 for

$Cp_2Ti(CH_2)(PMe_3)$, 0.5 for $Cp_2Ti(CH_3)_2$, $Cp^*{}_2Ti(CH_3)_2$, $Cp_2Ti(CH_2tBu)_2$, [nacnacTi(CHtBu)]OTf $TaCl(CH_2tBu)_2(CHtBu)$, $Cp_2Ta(CH_3)_2^+$ and 1.0 for $Ti(CH_2tBu)_4$ $[nacnacTi(CH_2tBu)_2]^+$, $TaCl_2(CH_2tBu)_3$, $TaCl(CH_2tBu)_4$, and $Cp_2Ta(CH_3)_3$.

The geometry optimization and the calculation of the NMR shielding tensors were carried out on molecular models that are identical to the experimental systems, with the exception of $[nacnacTi(CH_2tBu)_2][BArF_{24}]$ and $[Cp_2Ta(CH_3)_2][BF_4]$, where the counter anions are not introduced in the modelling.

Energies were calculated as single point calculations from the optimized structures, using GD3 dispersion corrections[28] and the SMD model[29] to account for the solvent (toluene).

3. Solid-State NMR Spectra

$Cp_2Ti(CH_3)_2$

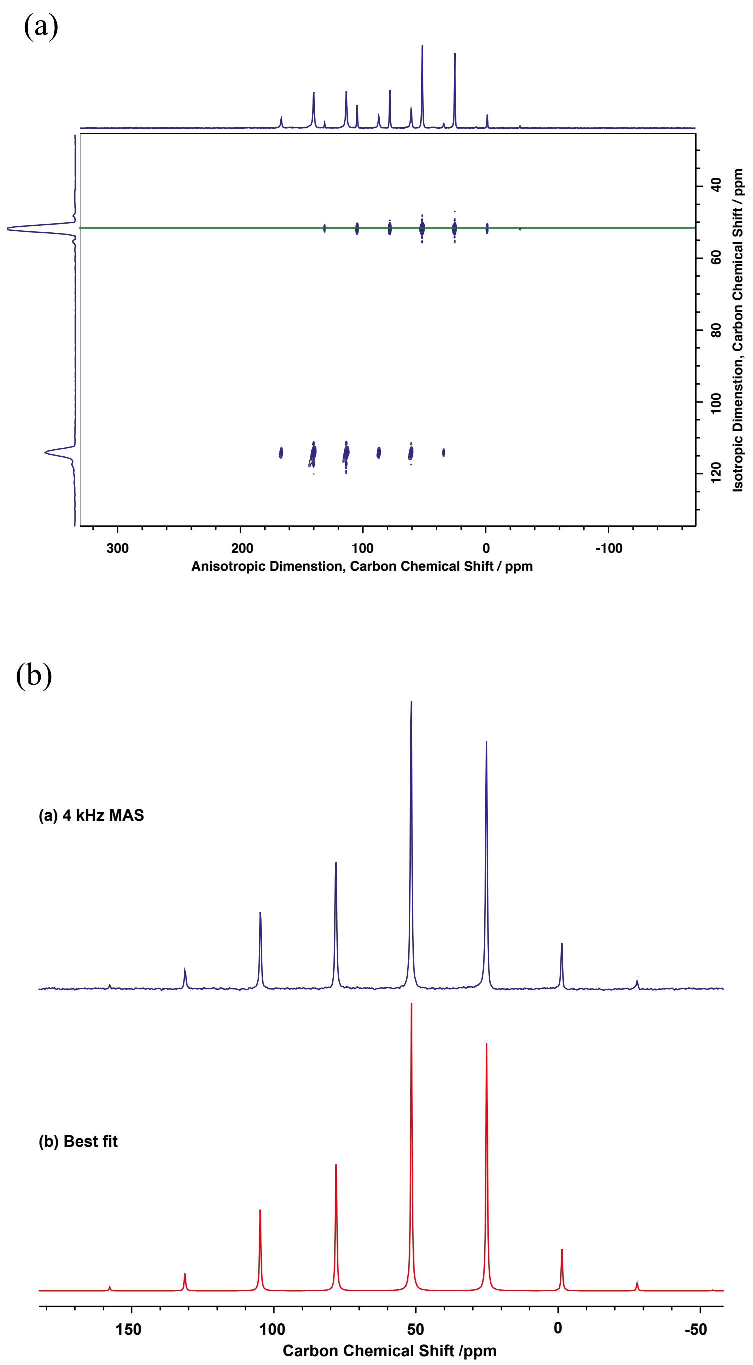

Figure S1. (a) The CP magic-angle turning[30] (CP-MAT, 14.1 T at 100 K) spectrum of **$Cp_2Ti(CH_3)_2$** at a spinning rate of 4 kHz. The contact time for CP was 0.5 ms, and the recycle delay was 1 s. 256 scans per *t1* increment and 91 *t1* increments were acquired. (b) Blue: the spectrum of the spinning side bands for the methyl carbon, which were obtained by slicing horizontally the CP-MAT spectrum at 52 ppm. Red: best-fit simulated spinning side bands of the corresponding carbon.

$Cp^*_2Ti(CH_3)_2$

(a)

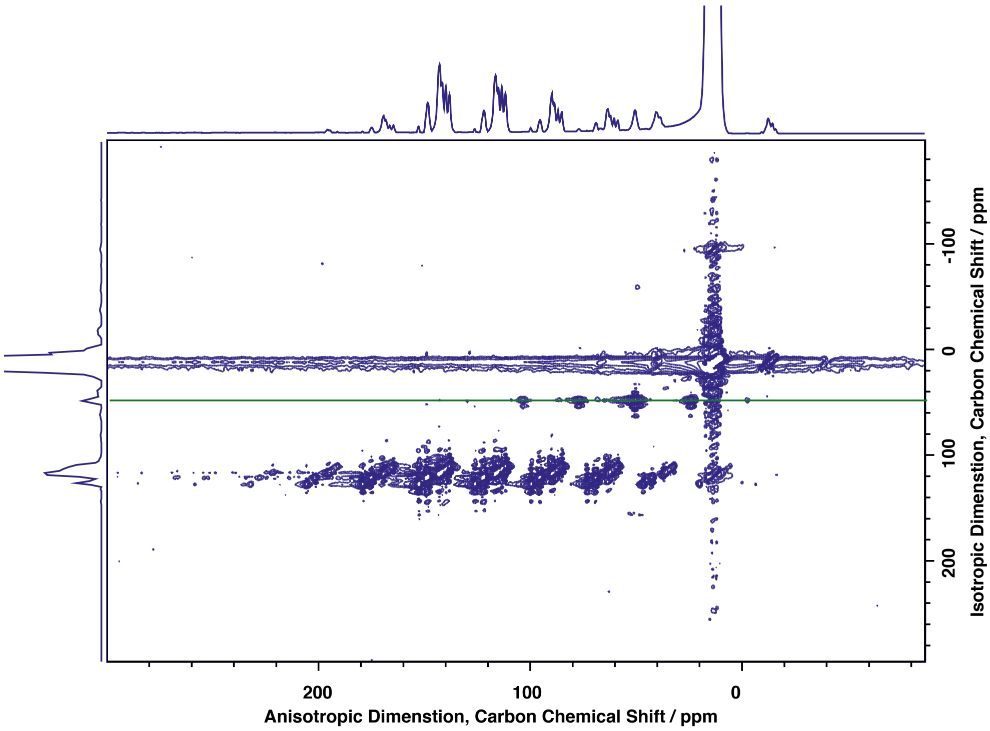

(b)

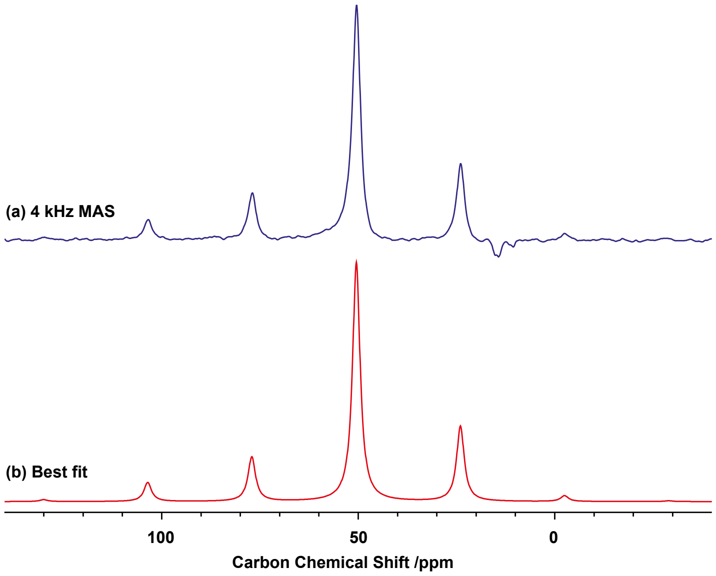

Figure S2. (a) The CP magic-angle turning[30] (CP-MAT, 14.1 T at 100 K) spectrum of **$Cp^*_2Ti(CH_3)_2$** at a spinning rate of 4 kHz. The contact time for CP was 0.5 ms, and the recycle delay was 1 s. 32 scans per *t1* increment and 441 *t1* increments were acquired. (b) Blue: the spectrum of the spinning side bands for the $\mathit{C}H_3$ carbon, which were obtained by slicing horizontally the CP-MAT spectrum at 50 ppm. Red: best-fit simulated spinning side bands of the corresponding carbon.

$Cp_2Ti(CH_2tBu)_2$

(a)

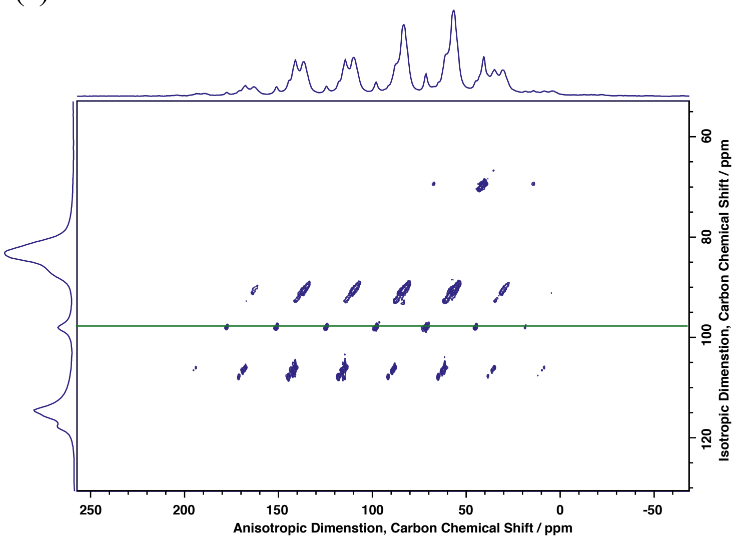

(b)

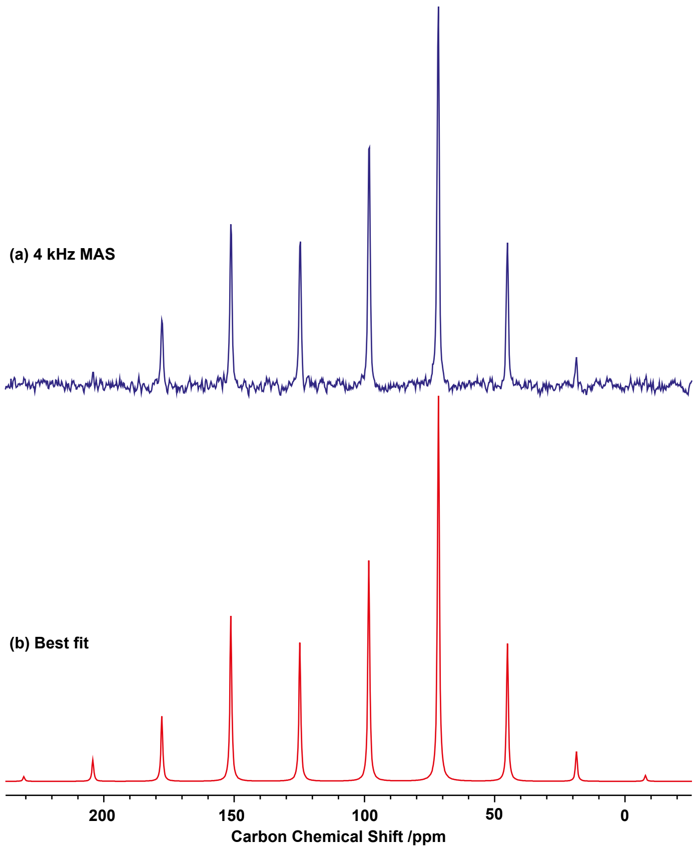

Figure S3. (a) The CP magic-angle turning[30] (CP-MAT, 14.1 T at 100 K) spectrum of **$Cp_2Ti(CH_2tBu)_2$** at a spinning rate of 4 kHz. The contact time for CP was 0.5 ms, and the recycle delay was 1 s. 256 scans per *t1* increment and 486 *t1* increments were acquired. (b) Blue: the spectrum of the spinning side bands for the $\mathit{C}H_2$tBu carbon, which were obtained by slicing horizontally the CP-MAT spectrum at 98 ppm. Red: best-fit simulated spinning side bands of the corresponding carbon.

$Ti(CH_2tBu)_4$

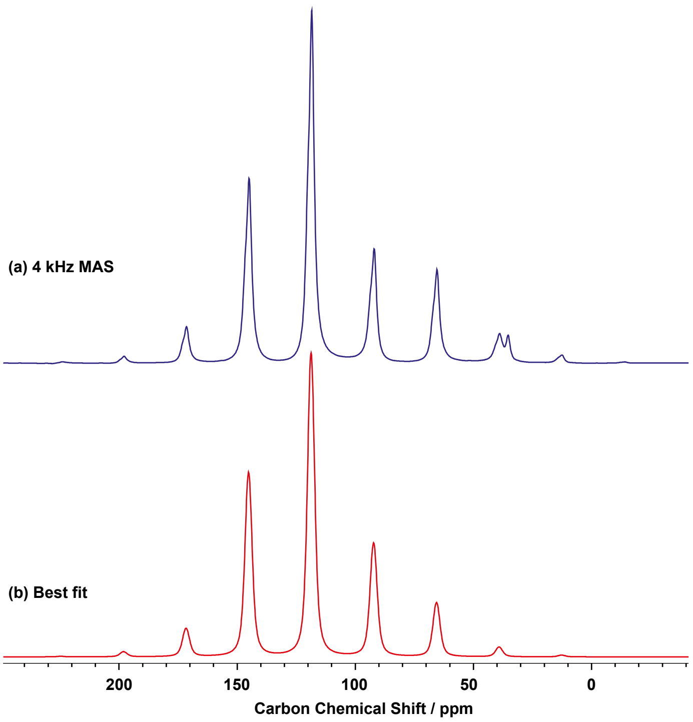

Figure S4. (a) Blue: The $^{13}C\{^1H\}$ CPMAS (14.1 T at 220 K) spectrum of **$Ti(CH_2tBu)_4$** at a spinning rate of 4 kHz. The contact time for CP was 0.5 ms, and the recycle delay was 0.5 s. (b) Red: best-fit simulated spinning side bands of the $\mathit{C}H_2$tBu carbons.

[nacnacTi(CH_2tBu)$_2$][BArF$_{24}$]

(a)

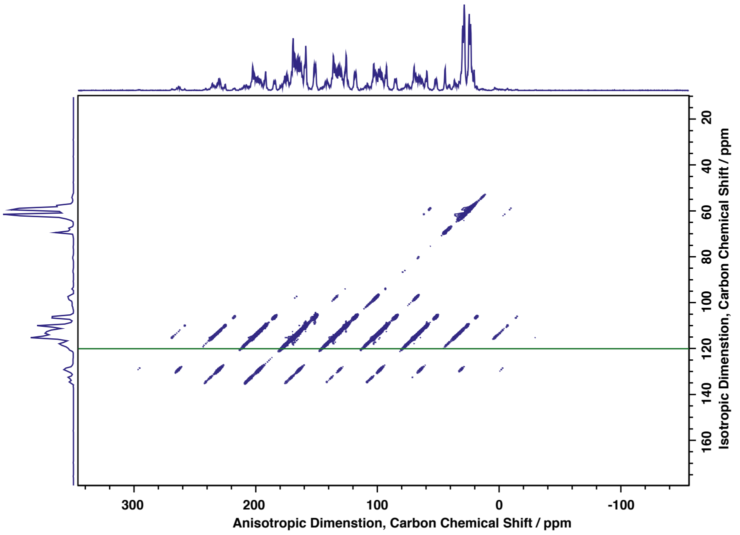

(b)

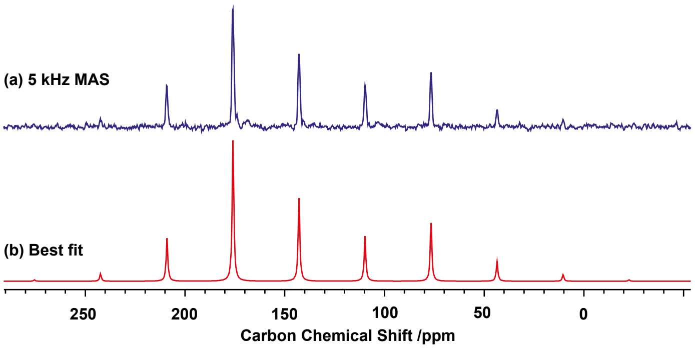

Figure S5. (a) The CP magic-angle turning[30] (CP-MAT, 14.1 T at 100 K) spectrum of **[nacnacTi(CH_2tBu)$_2$][BArF$_{24}$]** at a spinning rate of 5 kHz. The contact time for CP was 0.5 ms, and the recycle delay was 0.7 s. 160 scans per *t1* increment and 489 *t1* increments were acquired. (b) Blue: the spectrum of the spinning side bands for the *C*H_2tBu carbon, which were obtained by slicing horizontally the CP-MAT spectrum at 143 ppm. Red: best-fit simulated spinning side bands of the corresponding carbon.

The peak of the α-carbon falls close to aromatic signals from the nacnac-ligand and from BArF$_{24}$. It was assigned by a HETCOR experiment (HETeronuclear CORrelation spectroscopy), in which the ^{13}C signal at 143 ppm shows a crosspeak to the proton on the α-carbon at 2.9 ppm. The assignment is further confirmed by a very distinct CSA of that signal, differentiating it from the aromatic signals.

[nacnacTi(CHtBu)]OTf

(a)

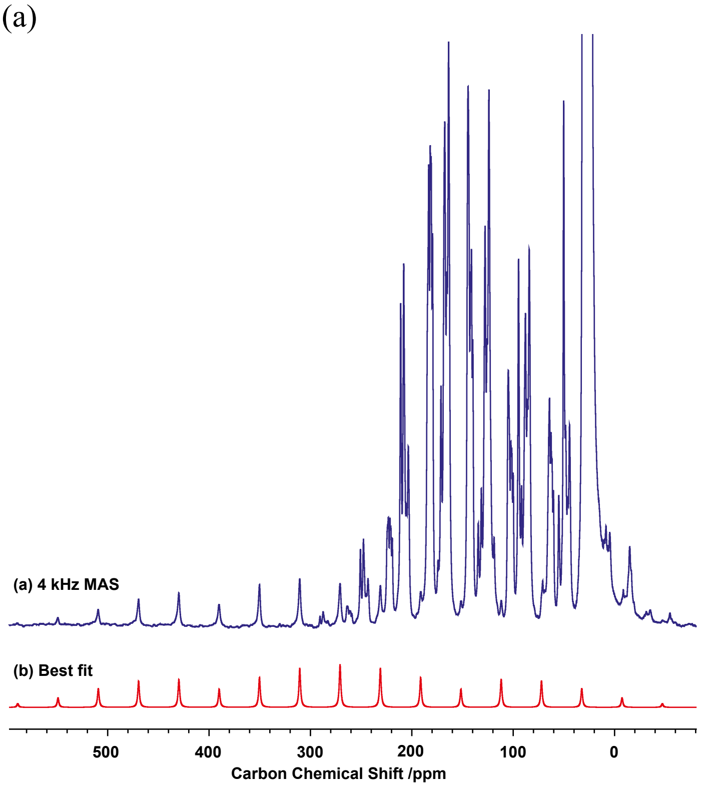

Figure S6. (a) Blue: The $^{13}C\{^1H\}$ CPMAS (9.4 T at room temperature) spectrum of **[nacnacTi(CHtBu)]OTf** at a spinning rate of 4 kHz. The contact time for CP was 2 ms, and the recycle delay was 2 s. (b) Red: best-fit simulated spinning side bands of the alkylidene carbon.

$TaCl(CH_2tBu)_4$

(a)

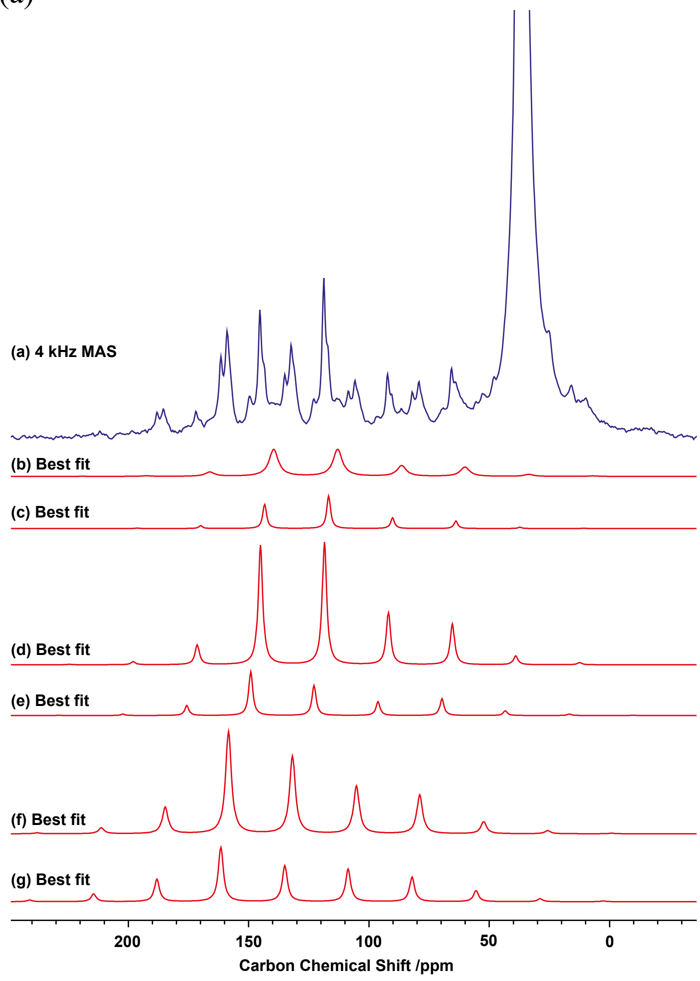

Figure S7. (a) Blue: The $^{13}C\{^1H\}$ CPMAS (14.1 T at 100 K) spectrum of $\mathbf{TaCl(CH_2tBu)_4}$ at a spinning rate of 4 kHz. The contact time for CP was 0.5 ms, and the recycle delay was 1 s. (b-g) Red: best-fit simulated spinning side bands of the $\mathit{C}H_2tBu$ carbons.

The solid state NMR spectrum of $\mathbf{TaCl(CH_2tBu)_4}$ shows 6 distinct sites. This contrasts the situation in solution NMR, where only two signals are observed in a 1:3 ratio (axial and equatorial carbon atoms respectively). Presumably, the presence of multiple conformers complicates the spectrum in the solid state, giving rise to α-carbons in various environments. All measured chemical shift tensors are given in the table below.

Table S1. Measured chemical shift tensors for $\mathbf{TaCl(CH_2tBu)_4}$.

site	δ_{iso}	δ_{11}	δ_{22}	δ_{33}
site 1	135	214	149	43
site 2	132	193	154	50
site 3	123	170	161	38
site 4	119	165	146	46
site 5	117	153	147	51
site 6	113	154	146	40

$TaCl_2(CH_2tBu)_3$

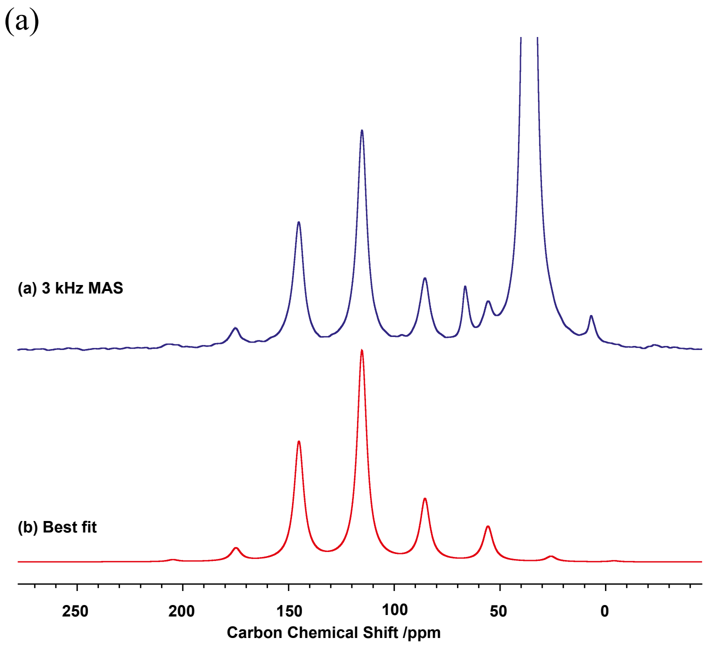

Figure S8. (a) Blue: The $^{13}C\{^1H\}$ CPMAS (9.4 T at room temperature) spectrum of **$TaCl_2(CH_2tBu)_3$** at a spinning rate of 3 kHz. The contact time for CP was 2 ms, and the recycle delay was 1 s. (b) Red: best-fit simulated spinning side bands of the *C*H_2tBu carbons.

$Cp_2Ta(CH_3)_3$

(a)

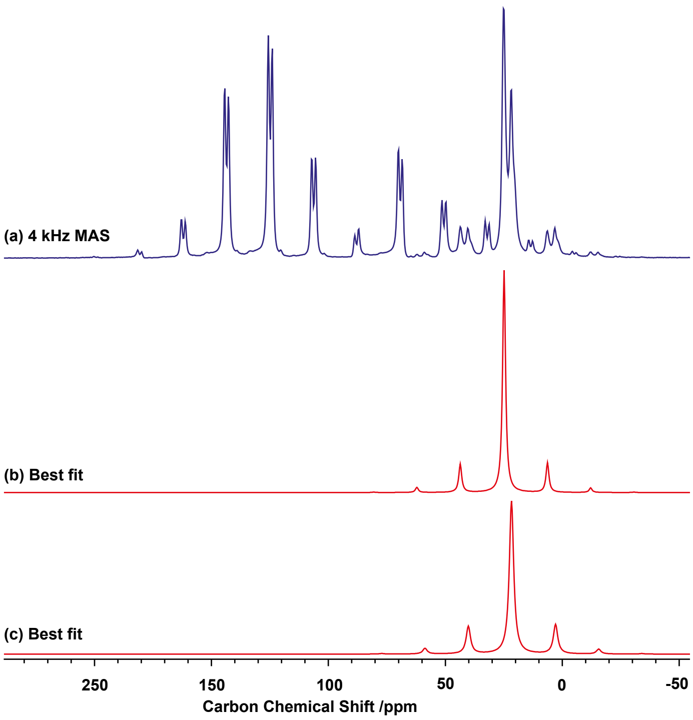

Figure S9. (a) Blue: The $^{13}C\{^1H\}$ CPMAS (14.1 T at 100 K) spectrum of **$Cp_2Ta(CH_3)_3$** at a spinning rate of 2.8 kHz. The contact time for CP was 0.5 ms, and the recycle delay was 1 s. (b-c) Red: best-fit simulated spinning side bands of the Me carbons.

$[Cp_2Ta(CH_3)_2][BF_4]$

(a)

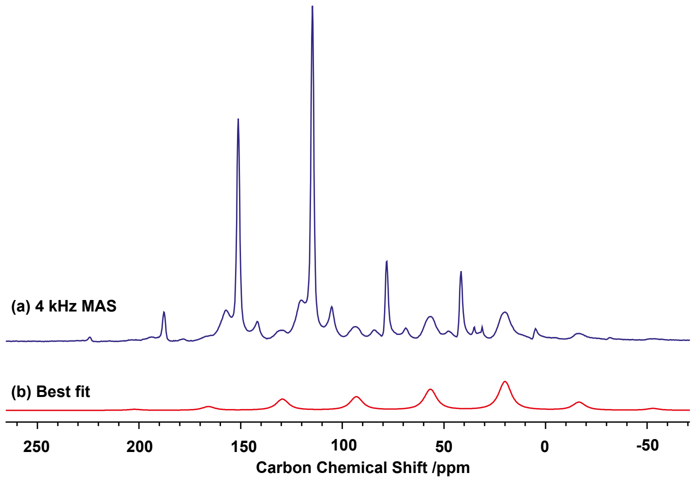

Figure S10. (a) Blue: The $^{13}C\{^1H\}$ CPMAS (14.1 T at 100 K) spectrum of **$[Cp_2Ta(CH_3)_2][BF_4]$** at a spinning rate of 5.5 kHz. The contact time for CP was 1 ms, and the recycle delay was 1 s. (b) Red: best-fit simulated spinning side bands of the Me carbons.

4. NMR Calculations

Table S2: Calculated shielding tensors (all values in ppm). Equatorial and axial ligands are indicated as eq and axial, respectively.

	σ_{iso}	σ_{11}	σ_{22}	σ_{33}	δ_{iso}	δ_{11}	δ_{22}	δ_{33}
**$Cp_2Ti(CH_3)_2$**	139	73	156	189	52	118	35	2
**$Cp^*{}_2Ti(CH_3)_2$**	140	77	165	179	51	114	26	12
**$Cp_2Ti(CH_2tBu)_2$**	105	33	131	152	86	158	60	39
**$Cp_2Ti(CH_2)$-PMe_3[a]**	-118	-564	36	174	309	754	155	17
**$[nacnacTi(CH_2tBu)_2]^+$**	52	-21	16	161	139	212	175	30
**nacnacTi(CHtBu)(OTf)**	-86	-422	-74	238	277	613	265	-47
**$Ti(CH_2tBu)_4$**	73	33	43	142	118	158	148	49
**$TaCl(CH_2tBu)_4$ (axial)**	49	-23	44	127	142	214	147	64
**$TaCl(CH_2tBu)_4$ (eq 1)**	62	2	42	141	129	189	149	50
**$TaCl(CH_2tBu)_4$ (eq 2)**	63	18	41	129	128	173	150	62
**$TaCl(CH_2tBu)_4$ (eq 3)**	64	32	29	133	126	159	162	58
**$TaCl(CH_2tBu)_2(CHtBu)$**	-73	-293	-120	193	264	484	310	-2
**$TaCl_2(CH_2tBu)_3$ (eq 1)**	61	20	39	126	130	171	152	65
**$TaCl_2(CH_2tBu)_3$ (eq 2)**	61	32	29	122	130	159	162	69
**$TaCl_2(CH_2tBu)_3$ (eq 3)**	74	30	60	131	117	161	131	60
**$Cp_2Ta(CH_3)_3$ (external)**	169	154	158	195	22	37	33	-4
**$Cp_2Ta(CH_3)_3$ (internal)**	168	142	176	184	23	49	15	7
**$[Cp_2Ta(CH_3)_2]^+$**	129	27	149	211	62	164	42	-20

[a] reported in [31]

Table S3: Diamagnetic and paramagnetic contributions to shielding (all values in ppm). Equatorial and axial ligands are indicated as eq and axial, respectively.

	σ_{11}		σ_{22}		σ_{33}	
	σ_{dia}	σ_{para}	σ_{dia}	σ_{para}	σ_{dia}	σ_{para}
**$Cp_2Ti(CH_3)_2$**	217	-144	222	-66	217	-28
**$Cp^*{}_2Ti(CH_3)_2$**	216	-139	222	-57	218	-39
**$Cp_2Ti(CH_2tBu)_2$**	224	-191	226	-95	224	-72
**$Cp_2Ti(CH_2)$-PMe_3[a]**	228	-792	250	-215	207	-34
**$Ti(CH_2tBu)_4$**	232	-199	231	-189	142	230
**$TaCl(CH_2tBu)_4$ (axial)**	228	-251	228	-184	230	-103
**$TaCl(CH_2tBu)_4$ (eq 1)**	224	-204	224	-185	230	-104
**$TaCl(CH_2tBu)_4$ (eq 2)**	225	-192	223	-194	230	-108
**$TaCl(CH_2tBu)_4$ (eq 3)**	224	-194	223	-164	229	-98
**$TaCl(CH_2tBu)_2(CHtBu)$**	242	-535	219	-338	257	-64
**$TaCl_2(CH_2tBu)_3$ (eq 1)**	224	-204	224	-185	230	-104
**$TaCl_2(CH_2tBu)_3$ (eq 2)**	225	-192	223	-194	230	-108
**$TaCl_2(CH_2tBu)_3$ (eq 3)**	224	-194	223	-164	229	-98
**$Cp_2Ta(CH_3)_3$ (external)**	212	-58	221	-63	214	-19
**$Cp_2Ta(CH_3)_3$ (internal)**	207	-65	213	-37	217	-33
**$[Cp_2Ta(CH_3)_2]^+$**	216	-190	225	-76	216	-6
**$[nacnacTi(CH_2tBu)_2]^+$**	232	-253	230	-214	233	-72

[a] reported in [31]

Table S4: NCS analysis of metal alkyl compounds – σ_{11} (most deshielded component; all values in ppm). Equatorial and axial ligands are indicated as eq and axial, respectively.

	σ_{dia}	σ_{para}	components of σ_{para}			
			σ(M-C)	σ(C-H)/π(M-C)	σ(C-C)/σ(C-H)	σ(C-H)
**$Cp_2Ti(CH_3)_2$**	217	-144	-127	-30	4	4
**$Cp^*{}_2Ti(CH_3)_2$**	216	-139	-118	-32	5	5
**$Cp_2Ti(CH_2tBu)_2$**	224	-191	-102	-20	-60	-7
**$Cp_2Ti(CH_2)$-PMe_3[a]**	228	-792	-478	-115	-90	-93
**$Ti(CH_2tBu)_4$**	232	-199	-103	-34	-49	0
**$TaCl(CH_2tBu)_4$ (axial)**	228	-251	-157	-31	-20	-1
**$TaCl(CH_2tBu)_4$ (eq 1)**	224	-223	-154	-17	-9	-3
**$TaCl(CH_2tBu)_4$ (eq 2)**	228	-210	-119	-19	-37	0
**$TaCl(CH_2tBu)_4$ (eq 3)**	227	-195	-123	-28	-21	2
**$TaCl(CH_2tBu)_2(CHtBu)$**	242	-535	-313	-71	-126	-11
**$TaCl_2(CH_2tBu)_3$ (eq 1)**	224	-204	-118	-15	-36	-4
**$TaCl_2(CH_2tBu)_3$ (eq 2)**	225	-192	-113	-16	-36	-4
**$TaCl_2(CH_2tBu)_3$ (eq 3)**	224	-194	-107	-13	-31	0
**$Cp_2Ta(CH_3)_3$ (ext. carbon)**	212	-58	-58	-4	-2	1
**$Cp_2Ta(CH_3)_3$ (int. carbon)**	207	-65	-66	-6	4	5
**$[Cp_2Ta(CH_3)_2]^+$**	216	-190	-174	-16	16	16
**$[nacnacTi(CH_2tBu)_2]^+$**	232	-253	-125	-47	-68	0

[a] reported in [31]

Table S5: NCS analysis of metal alkyl compounds – σ_{22} (all values in ppm). Equatorial and axial ligands are indicated as eq and axial, respectively.

	σ_{dia}	σ_{para}	components of σ_{para}			
			σ(M-C)	σ(C-H)/π(M-C)	σ(C-C)/σ(C-H)	σ(C-H)
**$Cp_2Ti(CH_3)_2$**	222	-66	-22	-14	-14	-13
**$Cp^*{}_2Ti(CH_3)_2$**	222	-57	-20	-15	-11	-7
**$Cp_2Ti(CH_2tBu)_2$**	226	-95	1	-38	-13	-34
**$Cp_2Ti(CH_2)$-PMe_3[a]**	250	-215	-2	-10	-105	-101
**$Ti(CH_2tBu)_4$**	231	-189	-123	-5	-22	-32
**$TaCl(CH_2tBu)_4$ (axial)**	228	-184	-106	-14	-26	-5
**$TaCl(CH_2tBu)_4$ (eq 1)**	228	-184	-106	-14	-26	-5
**$TaCl(CH_2tBu)_4$ (eq 2)**	228	-184	-106	-14	-26	-5
**$TaCl(CH_2tBu)_4$ (eq 3)**	228	-184	-106	-14	-26	-5
**$TaCl(CH_2tBu)_2(CHtBu)$**	219	-338	-172	-15	-71	-22
**$TaCl_2(CH_2tBu)_3$ (eq 1)**	224	-185	-131	-6	-8	0
**$TaCl_2(CH_2tBu)_3$ (eq 2)**	223	-194	-135	-8	-10	0
**$TaCl_2(CH_2tBu)_3$ (eq 3)**	223	-164	-117	-18	-1	-6
**$Cp_2Ta(CH_3)_3$ (ext. carbon)**	221	-63	-9	-18	-16	-17
**$Cp_2Ta(CH_3)_3$ (int. carbon)**	213	-37	-58	18	2	5
**$[Cp_2Ta(CH_3)_2]^+$**	225	-76	-20	-20	-20	-11
**$[nacnacTi(CH_2tBu)_2]^+$**	230	-214	-130	-46	-27	-8

[a] reported in [31]

Table S6: NCS analysis of metal alkyl compounds – σ_{33} (all values in ppm). Equatorial and axial ligands are indicated as eq and axial, respectively.

	σ_{dia}	σ_{para}	components of σ_{para}			
			σ(M-C)	σ(C-H)/π(M-C)	σ(C-C)/σ(C-H)	σ(C-H)
**$Cp_2Ti(CH_3)_2$**	217	-28	-34	-1	-1	7
**$Cp^*{}_2Ti(CH_3)_2$**	218	-39	-37	-2	-1	6
**$Cp_2Ti(CH_2tBu)_2$**	224	-72	-67	-5	-4	-2
**$Cp_2Ti(CH_2)$-PMe_3**[a]	207	-34	-50	0	4	14
**$Ti(CH_2tBu)_4$**	230	-88	-1	-22	-15	-24
**$TaCl(CH_2tBu)_4$ (axial)**	230	-103	-12	-34	-16	-20
**$TaCl(CH_2tBu)_4$ (eq 1)**	230	-89	-7	-22	-15	-21
**$TaCl(CH_2tBu)_4$ (eq 2)**	230	-101	-9	-28	-15	-23
**$TaCl(CH_2tBu)_4$ (eq 3)**	230	-97	-8	-26	-14	-24
**$TaCl(CH_2tBu)_2(CHtBu)$**	257	-64	38	-53	-17	0
**$TaCl_2(CH_2tBu)_3$ (eq 1)**	230	-104	-18	-26	-12	-26
**$TaCl_2(CH_2tBu)_3$ (eq 2)**	230	-108	-16	-28	-13	-26
**$TaCl_2(CH_2tBu)_3$ (eq 3)**	229	-98	-9	-24	-18	-22
**$Cp_2Ta(CH_3)_3$ (ext. carbon)**	214	-19	-61	21	19	3
**$Cp_2Ta(CH_3)_3$ (int. carbon)**	217	-33	-30	-13	4	11
**$[Cp_2Ta(CH_3)_2]^+$**	216	-6	-44	14	14	8
**$[nacnacTi(CH_2tBu)_2]^+$**	233	-72	7	-22	-14	-22

[a] reported in [31]

Table S7: Comparison of diamagnetic, paramagnetic and spin-orbit contributions to shielding (all values in ppm).

compound	$\sigma_{dia,iso}$ ($\sigma_{dia,11}$, $\sigma_{dia,22}$, $\sigma_{dia,33}$)	$\sigma_{para,iso}$ ($\sigma_{para,11}$, $\sigma_{para,22}$, $\sigma_{para,33}$)	$\sigma_{SO,iso}$ ($\sigma_{SO,11}$, $\sigma_{SO,22}$, $\sigma_{SO,33}$)
**$Cp_2Ti(CH_3)_2$**	224 (219, 222, 231)	-84 (-147, -70, -34)	-1 (-4, 0, 1)
**$Cp_2Ti(CH_2tBu)_2$**	238 (233, 238, 245)	-134 (-214, -103, -85)	-1 (-4, -1, 1)
**$[nacnacTi(CH_2tBu)_2]^+$**	241 (235, 240, 248)	-197 (-269, -232, -89)	-2 (-4, -3, 1)
**nacnacTi(CHtBu)(OTf)**	255 (238, 257, 272)	-339 (-681, -305, -32)	-2 (-7, -1, 1)
**$TaCl(CH_2tBu)_4$ (axial)**	241 (236, 240, 249)	-150 (-208, -141, -102)	-27 (-49, -31, -1)
**$[Cp_2Ta(CH_3)_2]^+$**	222 (212, 215, 240)	-79 (-149, -78, -11)	-9 (-32, 1, 5)

5. Graphical Representation of the Results of the NCS Analysis

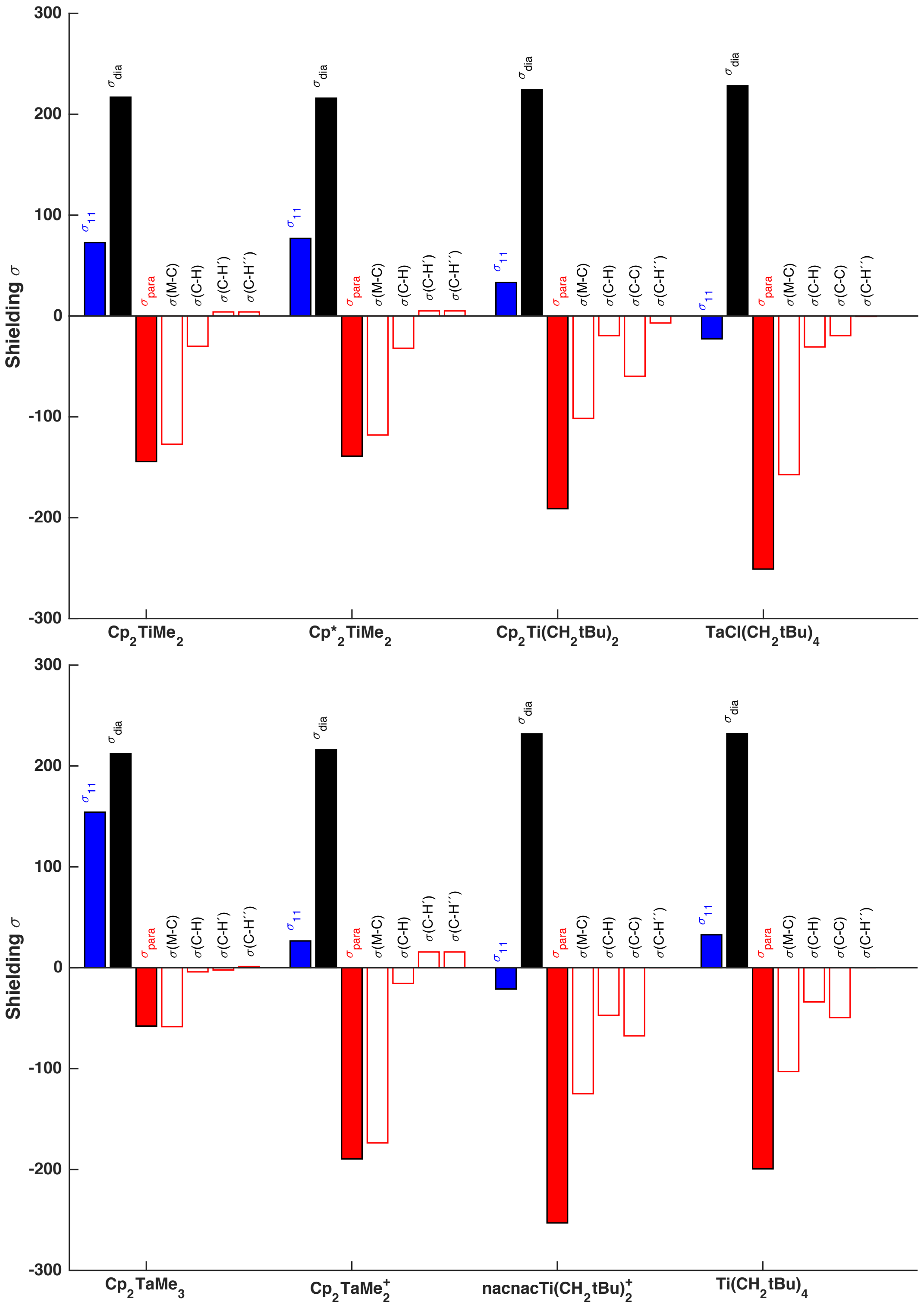

Figure S11. α-carbons of selected metal alkyl compounds – σ_{11} components (the axial carbon for $TaCl(CH_2tBu)_4$ and the external carbon for Cp_2TaMe_3 are shown).

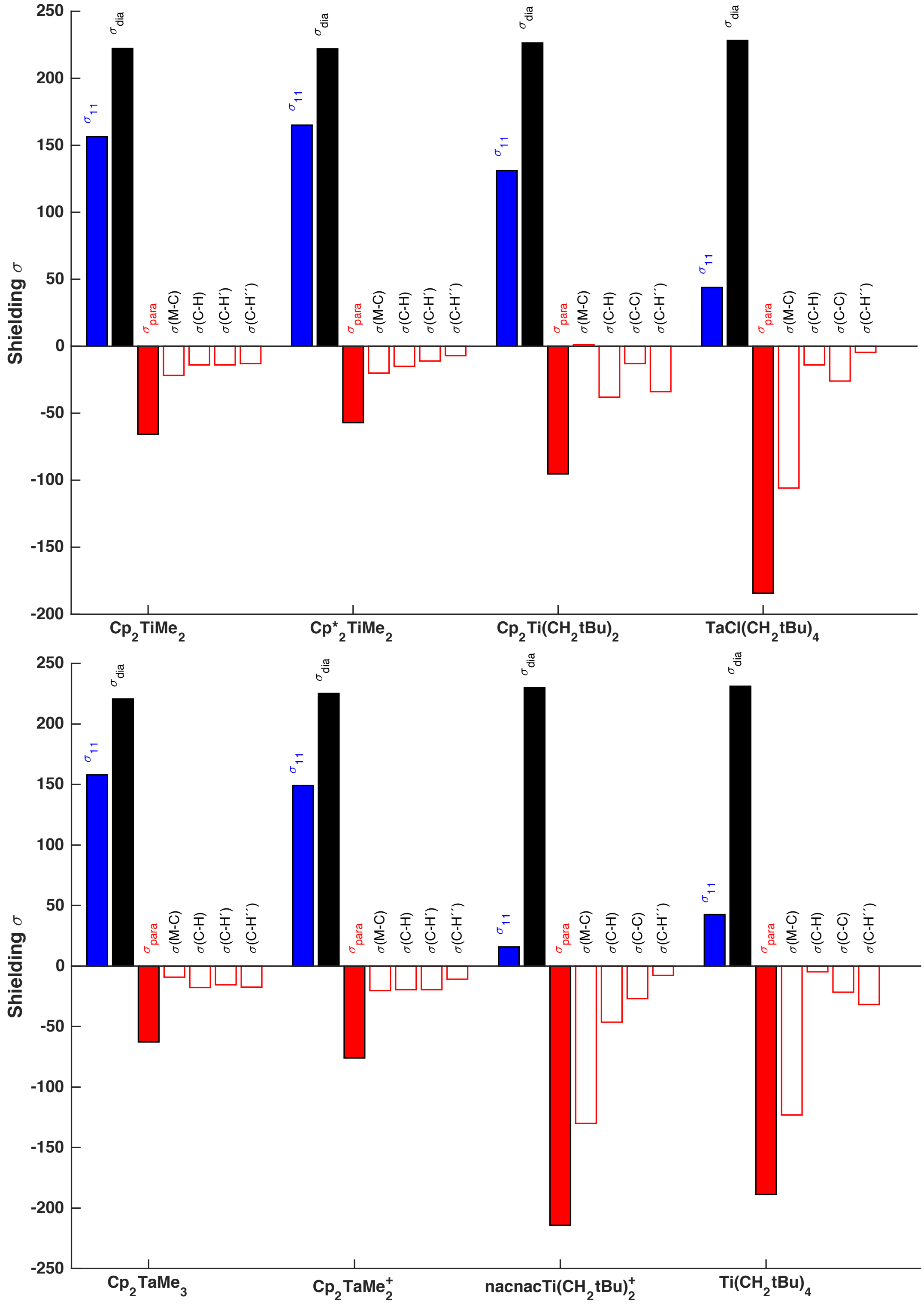

Figure S12. α-carbons of selected metal alkyl compounds – σ_{22} components (the axial carbon for $TaCl(CH_2tBu)_4$ and the external carbon for Cp_2TaMe_3 are shown).

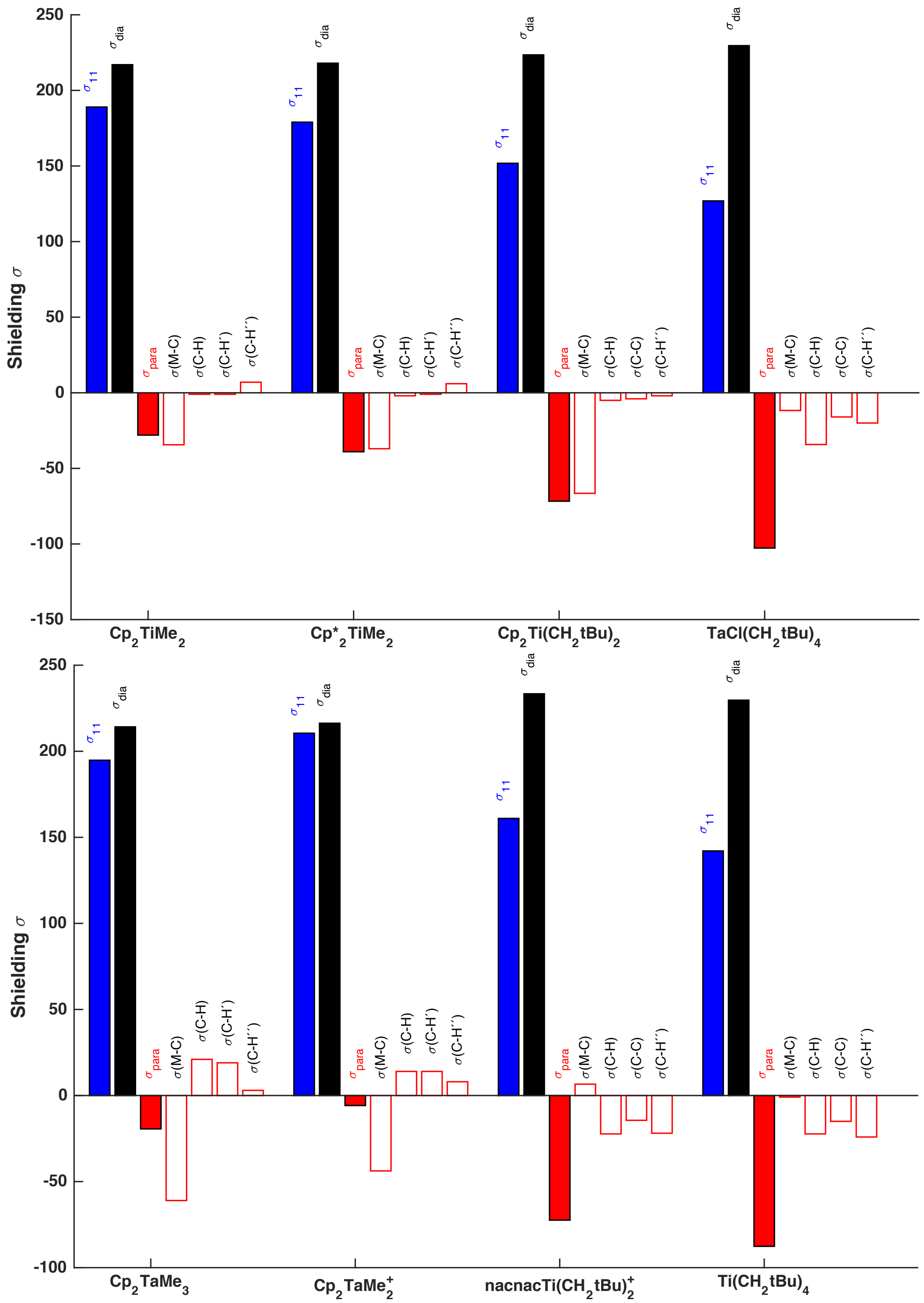

Figure S13. α-carbons of selected metal alkyl compounds – σ_{33} components (the axial carbon for $TaCl(CH_2tBu)_4$ and the external carbon for Cp_2TaMe_3 are shown).

6. Graphical Representations of the Calculated Shielding Tensors

Representation as polar plots of functions $\Sigma_{ij} r_i r_j \sigma_{ij}$. [26],[27]

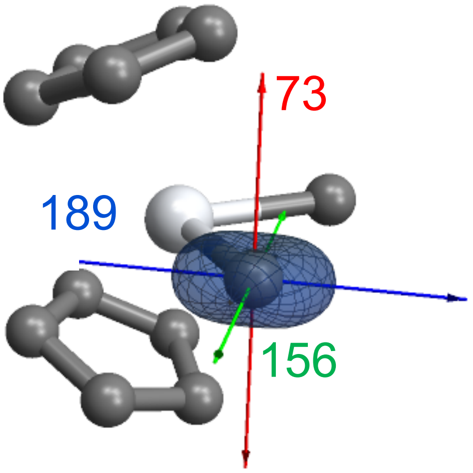

$Cp_2Ti(CH_3)_2$

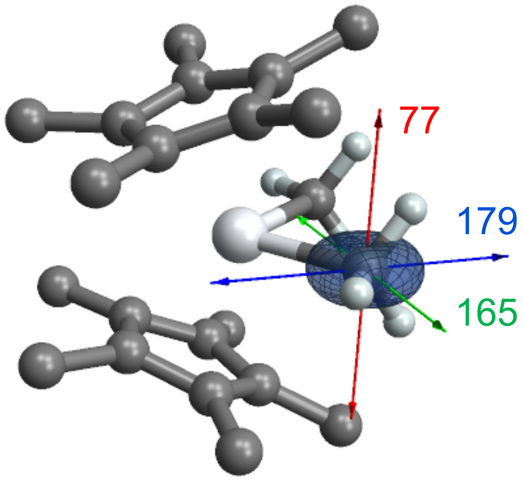

$Cp^*{}_2Ti(CH_3)_2$

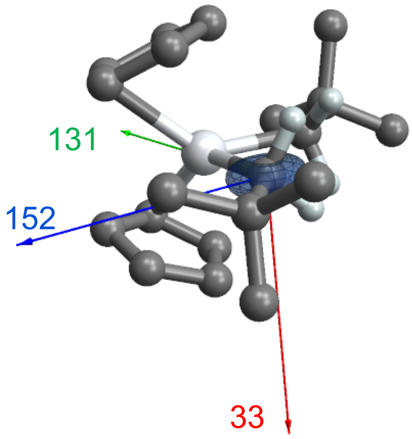

$Cp_2Ti(CH_{2t}Bu)_2$

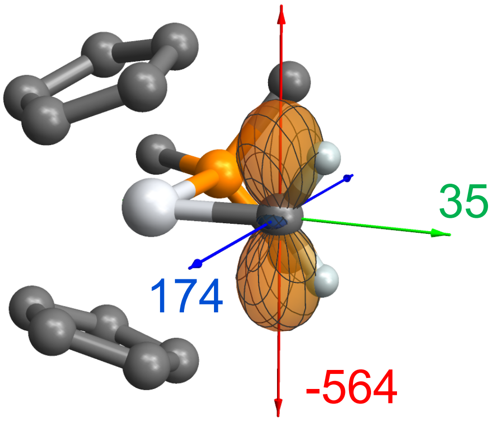

$Cp_2Ti(CH_2)$-PMe_3

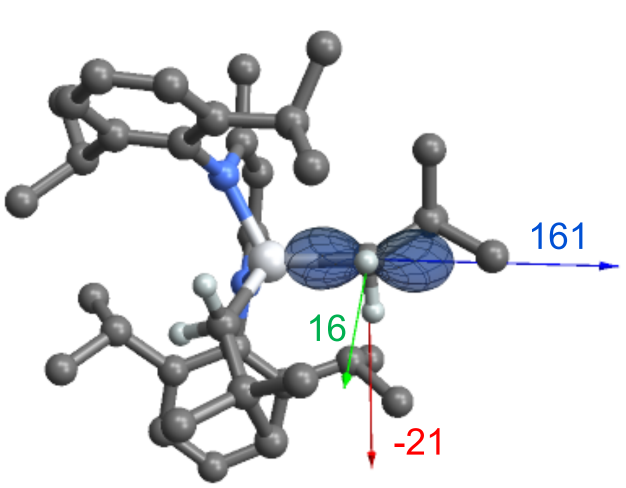

$[nacnacTi(CH_2tBu)_2^{+}]$

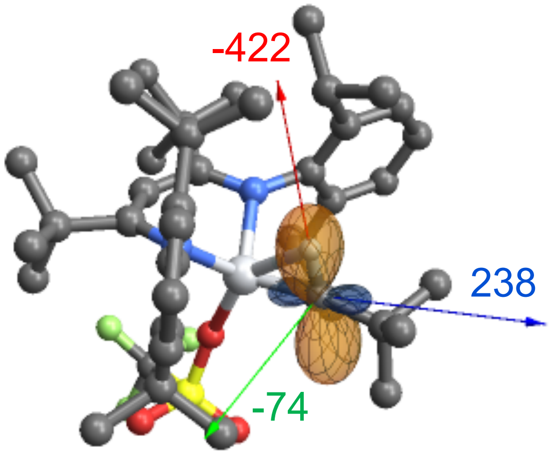

nacnacTi(CHtBu)(OTf)

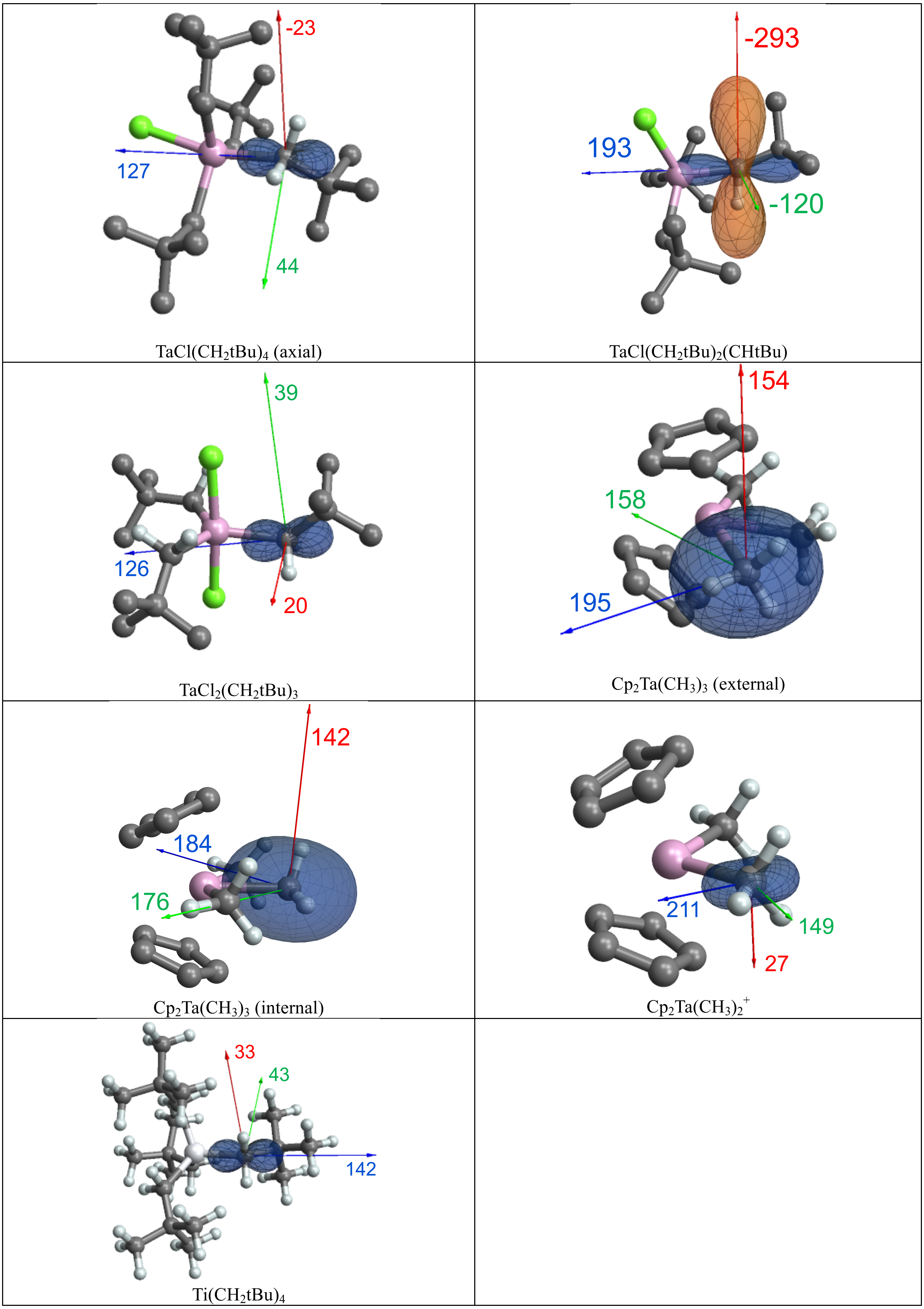

Figure S14. Representation of all calculated shielding tensors.

7. MO Diagrams of Representative Metal Alkyl Compounds

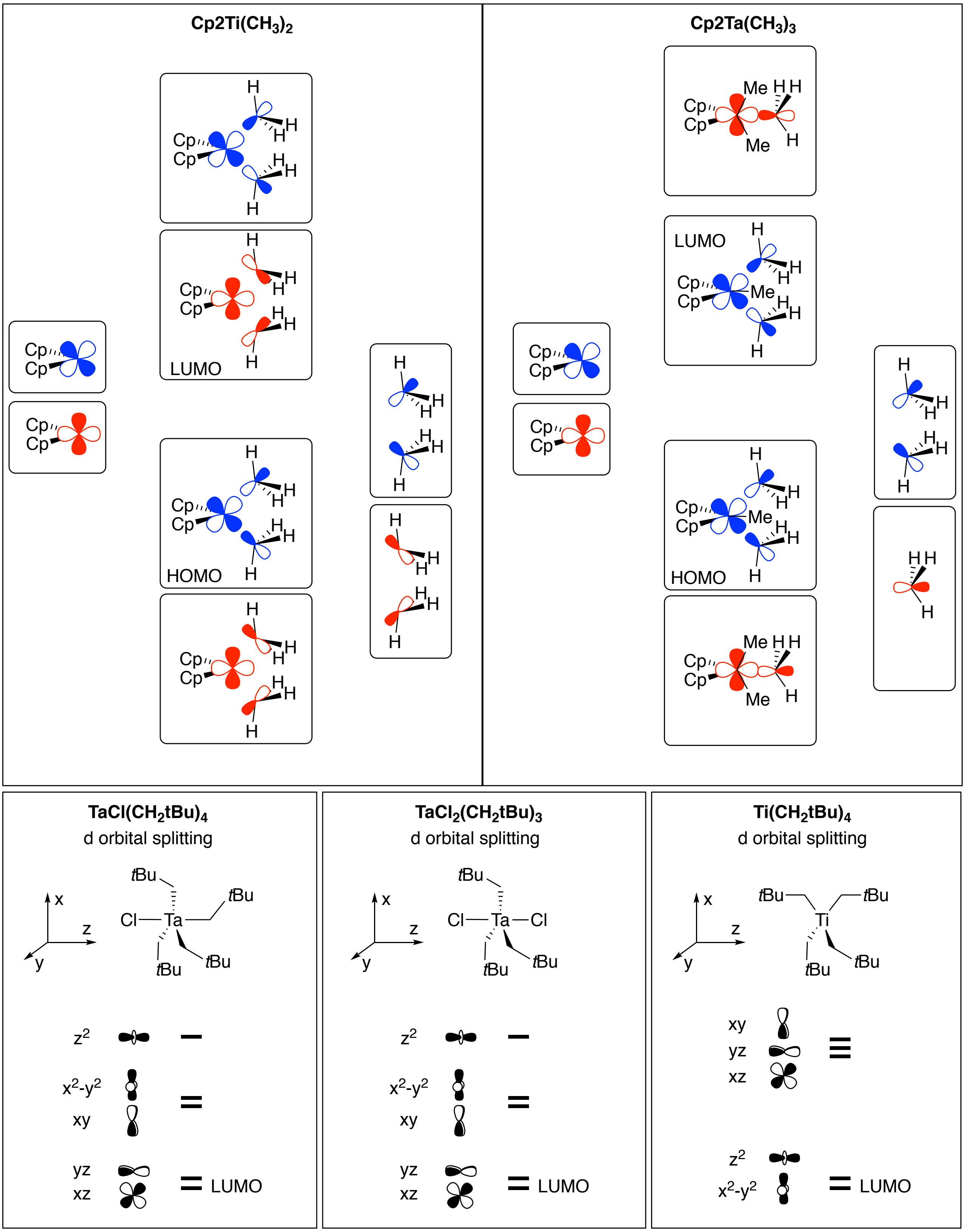

Figure S15. Frontier orbitals for $Cp_2Ti(CH_3)_2$, $Cp_2Ta(CH_3)_3$ and d orbital splitting in $TaCl(CH_2tBu)_4$.

8. Optimized Structures of all Calculated Species

Optimized Structures of all species are provided as .xyz files as supplementary material.

9. References

[1] Petasis, N. A.; Bzowej, E. I. *J. Am. Chem. Soc.* **1990,** *112*, 6392-6394.
[2] Mach, K.; Varga, V.; Hanuš, V. *J. Organomet. Chem.* **1991**, *415*, 87-95
[3] Meinhart, J. D.; Anslyn, E. V.; Grubbs, R. H. *Organometallics.* **1989,** *8*, 583-589.
[4] Basuli, F.; Bailey, B. C.; Watson, L. A.; Tomaszewski, J.; Huffman, J. C.; Mindiola, D. J. *Organometallics.* **2005,** *24*, 1886-1906.
[5] Cheon, J.; Rogers, D. M.; Girolami, G. S. *J. Am. Chem. Soc.* **1997,** *119*, 6804-6813
[6] Mowat, W.; Wilkinson, G. *J. Organomet. Chem.* **1972,** *38*, C35-C36.
[7] Schrock, R. R.; Fellmann, J. D. *J. Am. Chem. Soc.* **1978,** *100*, 3359-3370.
[8] Schrock, R. R.; Sharp, P. R. *J. Am. Chem. Soc.* **1978,** *100*, 2389-2399.
[9] Frisch, M. J.; Trucks, G. W.; Schlegel, H. B.; Scuseria, G. E.; Robb, M. A.; Cheeseman, J. R.; Scalmani, G.; Barone, V.; Mennucci, B.; Petersson, G. A.; Nakatsuji, H.; Caricato, M.; Li, X.; Hratchian, H. P.; Izmaylov, A. F.; Bloino, J.; Zheng, G.; Sonnenberg, J. L.; Hada, M.; Ehara, M.; Toyota, K.; Fukuda, R.; Hasegawa, J.; Ishida, M.; Nakajima, T.; Honda, Y.; Kitao, O.; Nakai, H; Vreven, T.; Montgomery, J. A.; Peralta, Jr. J. E.; Ogliaro, F.; Bearpark, M.; Heyd, J. J.; Brothers, E.; Kudin, K. N.; Staroverov, V. N.; Kobayashi, R.; Normand, J.; Raghavachari, K.; Rendell, A.; Burant, J. C.; Iyengar, S. S.; Tomasi, J.; Cossi, M.; Rega, N.; Millam, J. M.; Klene, M.; Knox, J. E.; Cross, J. B.; Bakken, V.; Adamo, C.; Jaramillo, J.; Gomperts, R.; Stratmann, R. E.; Yazyev, O.; Austin, A. J.; Cammi, R.; Pomelli, C.; Ochterski, J. W.; Martin, R. L.; Morokuma, K.; Zakrzewski, V. G.; Voth, G. A.; Salvador, P.; Dannenberg, J. J.; Dapprich, S.; Daniels, A. D.; Farkas, Ö.; Foresman, J. B.; Ortiz, J. V.; Cioslowski, J.; Fox, D. J. *Gaussian 09* (Gaussian, Inc., Wallingford CT, 2009) VERSION D.01.
[10] Adamo, C.; Barone, V. *J. Chem. Phys.* **1999**, *110*, 6158.
[11] Dolg, M.; Wedig, U.; Stoll, H.; Preuss, H. *J. Chem. Phys.* **1987**, *86*, 866.
[12] Andrae, D.; Haeussermann, U.; Dolg, M.; Stoll, H.; Preuss, H. *Theor. Chim. Acta* **1990**, *77*, 123.
[13] Martin, J. M. L.; Sundermann, A. *J. Chem. Phys.* **2001**, *114*, 3408.
[14] Jensen, J. *J. Chem. Theory Comput.*, **2014**, *10*, 1074.
[15] te Velde, G.; Bickelhaupt, F. M.; Baerends, E. J.; Fonseca Guerra, C.; van Gisbergen, S. J. A.; Snijders, J. G.; Ziegler T. *J. Comp. Chem.* **2001**, *22*, 931. Amsterdam Density Functional (ADF) Theoretical Chemistry Vrieje Universitet see http://www.scm.com/ VERSION 2014.
[16] van Lenthe, E.; Baerends, E. J.; Snijders, J. G. *J. Chem. Phys.* **1993**, *99*, 4597.
[17] van Lenthe, E.; Baerends E. J.; Snijders, J. G. *J. Chem. Phys.* **1994**, *101*, 9783.
[18] van Lenthe, E.; Baerends E. J.; Snijders, J. G. *J. Chem. Phys.* **1999**, *110*, 8943.
[19] van Lenthe, E.; Baerends, E. J.; Snijders, J. G. *J. Chem. Phys.* **1996**, *105*, 6505.
[20] van Lenthe, E.; van Leeuwen, R.; Baerends, E. J.; Snijders, J. G. *Int. J. Quant. Chem.* **1996**, *57*, 281.
[21] Glendening, E. D.; Badenhoop, J. K.; Reed, A. E.; Carpenter, J. E.; Bohmann, J. A.; Morales, C. M.; Landis, C. R.; Weinhold, F.; Theoretical Chemistry Institute, University of Wisconsin, Madison, WI, USA **2013**, http://nbo6.chem.wisc.edu/
[22] Bohmann, J. A.; Weinhold, F.; Farrar, T. C. J. *J. Chem. Phys.* **1997**, *107*, 1173.
[23] Autschbach, J.; Zheng, T. *Magn. Reson. Chem.* **2008**, *46*, S45.
[24] Autschbach, J. *J. Chem. Phys.* **2008**, *128*, 164112.
[25] Aquino, F.; Pritchard, B.; Autschbach, J. *J. Chem. Theory Comput.* **2012**, *8*, 598.
[26] Autschbach, J.; Zheng, S.; Shurko, R.W. *Concepts Magn Reson Part A.* **2010**, *36A*, 84.
[27] Zurek, E.; Pickard, C. J.; Autschbach, J. *J. Phys. Chem. C.* **2008**, *112*, 11744.
[28] Grimme, S.; Antony, J.; Ehrlich, S.; Krieg, H., *J. Chem. Phys.* **2010,** *132* (15), 154104.
[29] Marenich, A. V.; Cramer, C. J.; Truhlar, D. G., *J. Phys. Chem. B* **2009,** *113* (18), 6378-6396.
[30] Bax, A.; Szeverenyi, N. M.; Maciel, G. E. *J. Magn. Reson.* **1983**, *52*, 147.
[31] Gordon, C. P.; Yamamoto, K.; Liao, W.-C.; Allouche, F.; Andersen, R. A.; Copéret, C.; Raynaud, C.; Eisenstein, O., *ACS Cent. Sci.* **2017**, *3*, 759-768
